# Differentiation in fitness-related traits in response to elevated temperatures between leading and trailing edge populations of marine macrophytes

**DOI:** 10.1371/journal.pone.0203666

**Published:** 2018-09-13

**Authors:** Catarina F. Mota, Aschwin H. Engelen, Ester A. Serrao, Márcio A. G. Coelho, Núria Marbà, Dorte Krause-Jensen, Gareth A. Pearson

**Affiliations:** 1 Centro de Ciências do Mar (CCMAR), CIMAR, University of Algarve, Faro, Portugal; 2 Department of Global Change Research, IMEDEA (CSIC-UIB), Esporles, Spain; 3 Department of Bioscience, Aarhus University, Silkeborg, Denmark; 4 Arctic Research Centre, Aarhus University, Aarhus, Denmark; Griffith University, AUSTRALIA

## Abstract

The nature of species distribution boundaries is a key subject in ecology and evolution. Edge populations are potentially more exposed to climate-related environmental pressures. Despite research efforts, little is known about variability in fitness-related traits in leading (i.e., colder, high latitude) versus trailing (i.e., warmer, low latitude) edge populations. We tested whether the resilience, i.e. the resistance and recovery, of key traits differs between a distributional cold (Greenland) and warm (Portugal) range edge population of two foundation marine macrophytes, the intertidal macroalga *Fucus vesiculosus* and the subtidal seagrass *Zostera marina*. The resistance and recovery of edge populations to elevated seawater temperatures was compared under common experimental conditions using photosynthetic efficiency and expression of heat shock proteins (HSP). Cold and warm edge populations differed in their response, but this was species specific. The warm edge population of *F*. *vesiculosus* showed higher thermal resistance and recovery whereas the cold leading edge was less tolerant. The opposite was observed in *Z*. *marina*, with reduced recovery at the warm edge, while the cold edge was not markedly affected by warming. Our results confirm that differentiation of thermal stress responses can occur between leading and trailing edges, but such responses depend on local population traits and are thus not predictable just based on thermal pressures.

## Introduction

In the current context of climate change, predictions of future species ranges often involve research on thermal responses of species. Predictive approaches commonly assume that all individuals of a species have the same tolerance response to environmental stressors. However, previous ecological, evolutionary and conservation studies have questioned both the limits of adaptive evolution at range margins [1,2] and the importance of rear-edge refugial populations for biodiversity [3], implying that separate populations of a species can present distinct genetic and phenotypic features.

Peripheral populations are often expected to have low genetic diversity, due to reduced population size and genetic drift in fragmented or heterogeneous habitats, while reduced gene flow may also result in high differentiation between isolated edge populations [2]. Rapid environmental change is expected to trigger ecological and evolutionary responses [4–6], which will particularly affect peripheral populations at range edges restricted by climate-related factors. The relative performance of fitness-related traits in peripheral populations and their adaptive potential under predicted climate change scenarios, is poorly understood. The ‘abundant-center hypothesis’ in biogeographical ecology suggests that when gradients of abiotic stress shape species distributions the central range provides the most favourable conditions that progressively decline until the range limits, where the stress approaches levels that are too high to allow population persistence. However, little is known about how leading versus trailing edges may have different constraints on the ability to evolve given their distinct genetic diversities and past evolutionary history (e.g., [7]).

Recent empirical work, particularly in coastal marine systems [8–11], has challenged the ‘abundant center’ view, by highlighting how variation in environmental factors (e.g. temperature, tides, light exposure) produces a mosaic of stress factors and their intensities [9,12]. Steep vertical abiotic stress gradients have been widely described in intertidal habitats and are known to set the upper boundaries of species distributions [13–18]. These steep gradients may cause intertidal or shallow-water species to be especially sensitive to climatic change [19,20], making them early warning indicators of disturbance. Knowledge of how the ecology, genetics, and physiology of these intertidal or shallow-water organisms interplay with abiotic stress factors to shape their distribution range should help predict their responses to climate change.

Temperature is one of the main physical factors that determine the distribution of species. Biogeographic distributions of many macrophytes have been explained by temperature adaptation, phenotypic acclimation of performance and temperature tolerance [21]. Most studies that link temperature and species distribution have focused on mean conditions over time [22], whereas temperature extremes can substantially stress performance and restrict survival and reproduction [23]. Such effects are amplified in environments with large temperature fluctuations like in intertidal or shallow water habitats in the marine realm. Global climate change is characterised by both the change in mean variables and the increase in extreme events (e.g., heat waves) that strongly impact ecosystems and associated species [22,24]. Foundation species (also named bioengineer or structural species, for their ecological role in structuring ecosystems) in the intertidal/shallow subtidal regions of temperate coasts in the northern hemisphere frequently include brown algae of the genus *Fucus* or seagrasses of the genus *Zostera* when either a hard or soft substrate is available, respectively. Stands of these species can provide feeding, nursery habitat and shelter from some abiotic stressors, facilitating occupation by other species and thus enhancing diversity [25]. While the seagrass root and rhizome systems stabilize sediments, the macrophyte canopy alters the hydrodynamic environment (reviewed in [26]) and allows suspended particles to sediment (e.g., [27]). Hence, the resilience and persistence of foundation species in the face of climate change are of particular interest and importance.

As a unifying concept for ecological genomics, Franssen *et al*. [28] propose “transcriptomic resilience, analogous to ecological resilience, as an important measure to predict the tolerance of individuals and hence the fate of local populations in the face of global warming.” In this paper we will use the resilience definition by Hodgson *et al*. [29], encompassing both resistance to immediate physiological impacts and recovery after the stress exposure, to evaluate the tolerance of two foundation species to short-term water temperature increases. Resilience to desiccation and heat shock differ at the warm trailing edge in ecologically important populations of the genera *Fucus* and *Zostera* [7,28,30–33]. For instance, some warm edge populations of *F*. *serratus* poor in genetic diversity were less resilient to desiccation and heat-shock than central ones in contrast with the expectation from selective pressures [7]. Edge differences in heat-shock response were also detected in Arctic *F*. *distichus* [31]. In the genus *Zostera*, southern and central populations did not differ in gene expression during a simulated heat wave but diverged during the recovery phase [28,33]. Overall, previous evidence suggests that functional traits may be significantly altered in marginal habitats in unpredictable ways, as local population traits (e.g., low diversity reducing ability to evolve) may interact against selective pressures [7]. Therefore, changing climate conditions may threaten small, fragmented and/or marginal populations because of inherently reduced fitness and lower adaptive capacity.

In this study, we tested the hypothesis that resilience of foundation species to warming differs between thermally opposite range edges. We investigated functional differentiation in response to rising seawater temperatures between populations from the warm (Portugal) and cold (Greenland) limits of *F*. *vesiculosus* and *Z*. *marina*. Under common experimental conditions, both photosynthetic efficiency, a key fitness-related trait, and the gene expression of heat shock proteins (HSP) were tested in response to elevated seawater temperatures. Our results show differentiation of a thermal stress response between leading and trailing edges and highlight the importance of local population responses for predictions of biodiversity changes in response to warming.

## Methods

### Model species and sampling

*Fucus vesiculosus* is a dioecious key foundation brown algal species, important from intertidal rocky shores to tidal marshlands. It occurs on the intertidal zone from northern Norway to Morocco along the eastern Atlantic, but extends also into some brackish and subtidal environments such as the Baltic and the White Sea. Along western Atlantic shores, it is found in similar rocky/marshland habitats ranging from Arctic Canada/Greenland to North Carolina (USA). Leading edge samples were collected in Greenland in front of the Greenland Institute of Natural Resources in Nuuk (64°11'46''N, 51°42'11''W), on August 21, 2010 (daylength 15:45 h), with permission of the Greenland Institute of Natural Resources. Trailing edge populations were sampled in Alcochete, Portugal (38°45'35''N, 8°57'14''W), on August 25, 2010, when daylength lasted 13:18 h. Fronds were haphazardly chosen about 0.5 m apart and cut from different individuals in the field and immediately prepared for transport (see below). Mean seawater surface temperatures range from a long-term minimum of -1.8 to a maximum of 6.5°C in Nuuk, and in Alcochete from 13.9 to 18.9°C (Seatemperature.org). At Nuuk, the annual air temperature range in 2010 was from -15.6 to 21.7°C [34].

*Zostera marina*, or eelgrass, is a marine angiosperm and the dominant seagrass in temperate shallow coastal waters of the northern hemisphere with a distribution on both Atlantic and Pacific coasts [35,36]. Reproduction takes place both clonally through vegetative growth and sexually by seeds. In Europe, *Z*. *marina* has its southern Atlantic limit in the Ria Formosa lagoon, Portugal, extending from the Mediterranean to the Arctic Circle [35]. Leading edge samples were collected in Kobbefjord near Nuuk, Greenland (64°09'40''N, 51°33'22''W), on August 21, 2010, at a day length of 15:43 h, whereas trailing edge populations were sampled during low tide from Culatra Island in the Ria Formosa lagoon, South Portugal (36°59'51''N, 7°49'41''W), on August 22, 2010, when daylength was 13:45. During sampling at Kobbefjord, salinity was 34 psu and seawater was 9.0°C, the annual seawater temperature ranges from -1 to +10 in 2012–2013 [37]. Mean seawater surface temperature in the lagoon ranges from 14.7°C to 23.4°C (Seatemperature.org) but can locally reach up to 36°C in shallow water [38]. Shoots were collected one step (±0.5 m) apart. Edge populations of *Z*. *marina* (from both Greenland and Portugal) generally have lower genetic and clonal diversity per population than in central regions [39,40]. For regional genetic diversity, pooling all populations per region, the northern (leading) edge is less diverse than central regions or the trailing edge (southern Iberia) [39], likely reflecting founder effects of postglacial recolonization, whereas the southern edge has the remnants of ancient distinct gene pools.

All macrophytes, about 65 fronds (± 5 cm) or shoots per species x location combination, were transported alive wrapped in paper towels moist with seawater, inside Ziploc bags in cool boxes with cooling elements, as commonly used for marine macrophytes (e.g., [41]). Those from Portugal were returned to the laboratory within three hours of collection, whereas macrophytes from Greenland were transported in four days. Up on arrival in Faro, tissues were in very good condition and those few in doubtful condition were discarded. Sixty *Fucus* apical tips of about 5 cm or *Z*. *marina* shoots (henceforth “tissue”) were cut and cultured in 5 L tanks of aerated and filtered natural seawater at 10°C for three days before the experiment. Tissue samples were maintained in water baths inside a walk-in climate chamber (Aralab 20000 EHF), under ambient day length conditions and photosynthetic photon flux density (PPFD) of 200 μmol m^-2^s^-1^, provided by a combination of Osram L58W/840 TLs and Kolorarc KRC400/T/H/960E40 HQI lamps. The temperature variation within each treatment was kept within ±0.3°C. Three-quarters of the seawater volume was replaced after two days during the acclimation period.

### Heat shock experiment and physiological measurements

After acclimation, macrophyte responses to temperature stress were tested through exposure of 5 replicates of each species x location x temperature x time combination to elevated seawater temperature scenarios that could typically occur during tidal cycles. For the experiments, the tissue was exposed to seawater of 18, 24, 28 or 32°C for three hours (typical low tide exposure), at a PPFD of 200 μmol m^-2^s^-1^, after which it was transferred back to acclimation conditions for recovery during 21 h. Control treatments were kept at 10°C under identical conditions as the other treatments. The used light intensity was a compromise between the usually used low saturating light level, not very realistic for natural conditions and the much higher stressing light conditions during a summer midday low tides where light levels can reach 1500 μmol m^-2^s^-1^ (GAP, pers. Observation). Photoinhibition of PSII maximum quantum yield (*Fv/Fm*) was measured on two positions from each of five replicate individuals per treatment with a chlorophyll fluorometer (Junior PAM, Walz, Germany) immediately after the 3 h exposure phase (hereafter 3h HS) and 21 h recovery phase (hereafter 24h Recovery). *Fv/Fm* scales directly with the quantum efficiency of PSII photochemistry [42], and its reduction from maximal values (about 0.8 in seagrasses, slightly lower in brown algae) is a sensitive and rapid screening tool for stress responses. The fast and simple measurement of *Fv/Fm* makes it a very useful stress indicator despite its limitations. Short-term impacts on *Fv/Fm* (measured immediately after the 3 h HS) reflect photodamage but also the effects of protective mechanisms (photoprotection). After recovery in constant acclimation conditions, the long-term impacts (*Fv/Fm* decrease) are assumed to reflect only lasting photodamage from the high temperature exposure. Resistance to the heat-stress is considered here as the ability to withstand the elevated temperatures without visible physiological impacts (at 3h HS), while recovery is the absence of long-term detrimental effects of high temperature exposure (after 24h). Tissue subsamples (two per individual) used in the physiological measurements were dark adapted for 5 min in leaf clip holders, and the remaining tissue immediately frozen in liquid nitrogen and stored at -80°C for RNA extraction. Baseline *Fv/Fm* values (10°C), i.e., controls, were measured at 3 and 24 h and values were generally similar (Table F in S1 File). Controls were manipulated as for temperature treatments but maintained at acclimation temperature. The heat shock experiments were conducted in programmed thermostatically controlled water baths within a walk-in climate chamber (Aralab 20000 EHF). *Fv/Fm* data were normalised within each population and sampling time (divided by the mean values of 10°C controls, n = 5 individuals) to account for intrinsic differences and to allow comparisons of stress resilience across populations and species. The experimental design tested for differentiation between species (fixed factor, two levels), marginality (Edge, fixed factor, two levels), and temperature (fixed factor, five levels).

For the gene expression study of *Fucus vesiculosus*, tissue samples were collected before PAM measurements, flash frozen in liquid nitrogen and stored at -80°C. Total RNA was extracted, DNase-treated and purified as described previously [7,43], from triplicate samples from each edge (Greenland or Portugal), treatment phase (3 h HS or 24 h Recovery) and temperature (10, 24, 28 or 32 ± 0.3°C) combination. RNA integrity was assessed by gel electrophoresis for every sample and only high-quality RNA was used for subsequent analysis by qPCR. Total RNA (500 ng) was reverse-transcribed with SuperScript III RT (Invitrogen) and oligo-dTprimer in two independent reactions that were then pooled. Quantitative PCR (qPCR) was performed in duplicate on an iCycler iQ Detection System (BioRad), as described previously [44]. The resulting files were analysed using iQ5 software (BioRad), with manual threshold settings and efficiency correction. The resulting individual expression values, normalised to the geometric average of three reference genes (EF1, Sumo3, and Tubulin) were used for further analysis.

The seven selected transcripts were identified from EST libraries for heat and desiccation stress in *F*. *vesiculosus* and *F*. *serratus* [45] and belong to four functionally diverse HSP families: HSP90, HSP70, HSP20 and ClpB/HSP100. One HSP70 sequence is a plastid-encoded DnaK (hs447), while the other (hs696) is a putative HSP70 cytosolic chaperone [46]. HSP20-2 is a small HSP (sHSP), a family which functions as ATP-independent chaperones preventing aggregation of misfolded proteins. sHSPs are typically only induced upon stress, quickly stabilizing denatured and aggregated proteins until they can be delivered to other chaperones for subsequent refolding by ATP-dependent chaperones such as the DnaK system or ClpB/DnaK [47]. The Clp_7H01 transcript is a member of the casein lytic proteinase/ heat shock protein 100 (Clp/Hsp100) family with sequence similarities to a ClpB chaperone, involved in delivering protein aggregates to other chaperones for refolding. As the three HSP90 transcripts had limited similarity to database sequences (of any organisms), their subcellular localization remains unknown.

Data were analysed using the non-parametric PERMANOVA module [47,48,49] within Primer 6 software [50] following Pearson *et al*. [7]. For each species and time (immediately after the heat shock or after recovery) the effects of Edge and Temperature were tested. Tests (distance-based homogeneity of dispersion, main effects and pair-wise) were made on a data matrix of Euclidean distances using 999 unrestricted permutations of raw data. As at the leading edge marine macrophytes are genetically extremely uniform compared to rear edge populations, edge effects were studied using two contrasting species rather than replication population within edges that are genetically identical [39]. Moreover, similar experiments including site replication did not detect differences in stress response among populations [51].

## Results

### Photosynthetic efficiency

**Fig 1:**
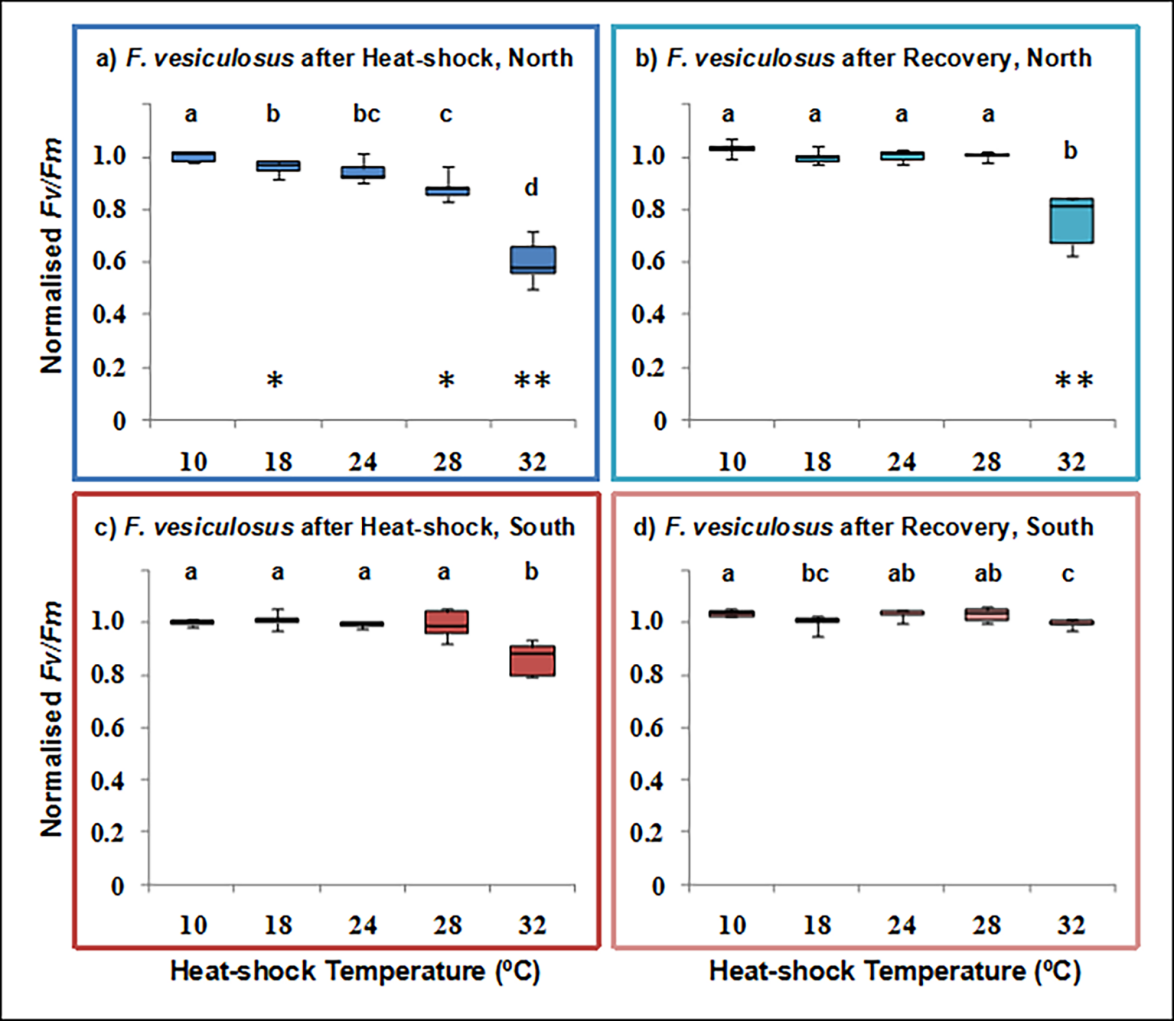
Photosynthetic temperature response of *Fucus vesiculosus*. Normalised maximum quantum yield (Fv/Fm) for the alga *Fucus vesiculosus* from the leading (Greenland, blue) and rear (Portugal, red) edge of distribution, directly after a 3 hour exposure to control (10°C) or elevated temperatures (18, 24, 28 and 32°C) and after a 21 hour recovery at control temperatures. Boxplot horizontal lines show the median, boxes show the 50% quartiles, and the error bars display the range of the data (n = 5). Asterisks show significant pair-wise differences between edges (* p<0.05; ** p<0.01) and different letters indicate significant pair-wise differences (p<0.05) between temperatures.

**Fig 2:**
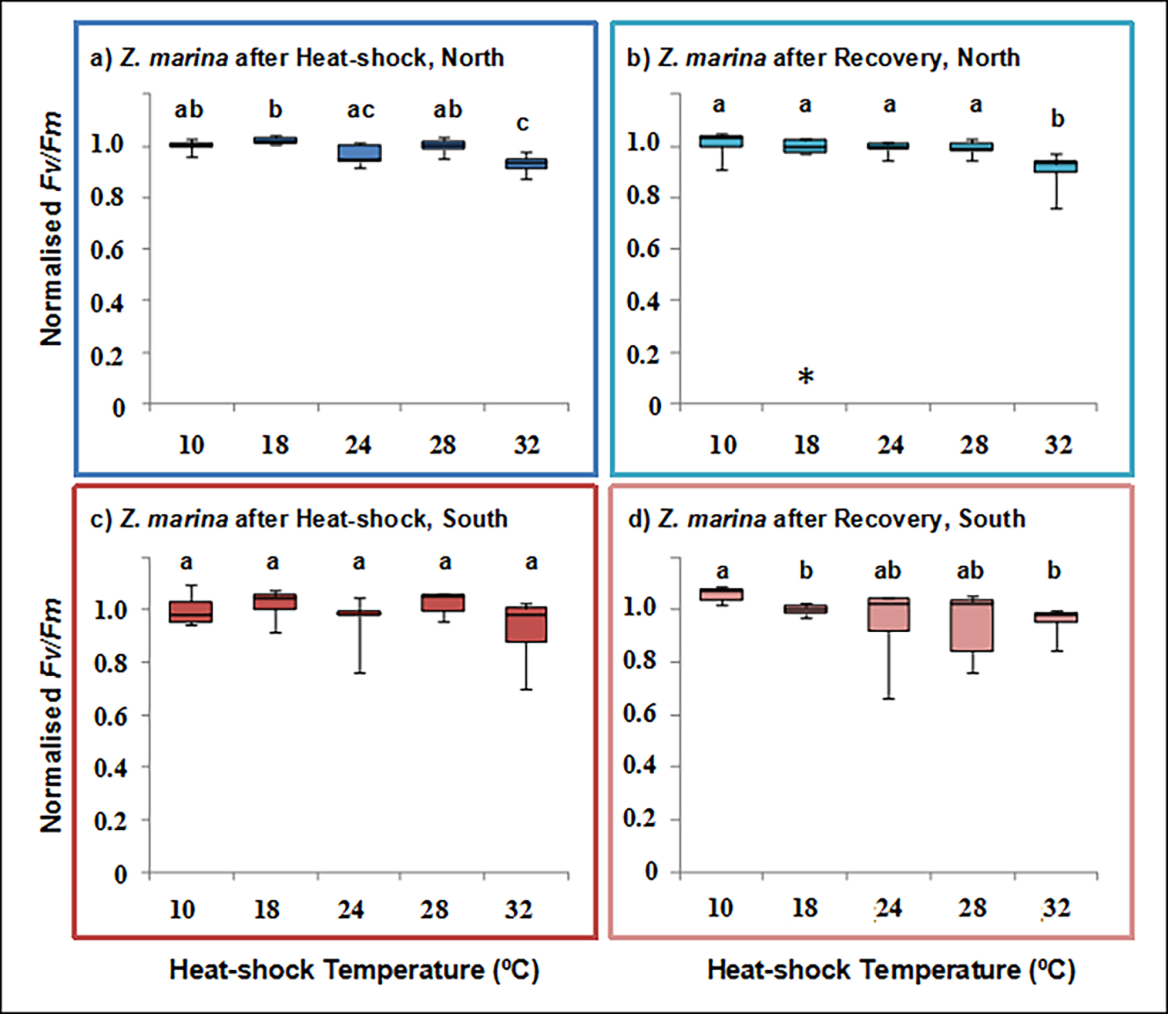
Photosynthetic temperature response of *Zostera marina*. Normalised maximum quantum yield (*Fv/Fm*) for the seagrass *Zostera marina* from the leading (Greenland, blue) and trailing (Portugal, red) edge of distribution, directly after a 3 hours exposure (HS) to control (10°C) or elevated temperatures (18, 24, 28 and 32°C) and after recovery at control temperatures. Boxplot horizontal lines show the median, boxes show the 50% quartiles, and the error bars display the range of the data (n = 5). Asterisks show significant pair-wise differences between edges (* p<0.05) and different letters indicate significant pairwise differences between temperatures (p<0.05).

The maximum quantum yield of PSII (*Fv/Fm*) was differentially impacted by exposure to elevated temperatures both between edge populations and between species, as assessed by the decrease in normalized *Fv/Fm* (Figs 1 and 2). Edge effects were therefore species dependent.

The response of *Fv/Fm* to 3 h heat shock (HS) differed markedly between the warm southern (trailing) and the cold northern (leading) edge populations of *Fucus vesiculosus*. While the northern edge population showed a progressive decline from 18°C onwards, *Fv/Fm* in the southern one remained unaffected by 3 h HS up to 28°C and decreased significantly (14%) only at 32°C. As a consequence, *Fv/Fm* at HS was significantly lower (ranging from 4–40%) in the northern population at all temperatures except 24°C (Fig 1A and 1C; Table 1 and Table A in S1 File). Population-specific differences in recovery were revealed at the highest HS temperature (32°C, Fig 1B and 1D). Hence, while statistical tests indicated that *Fv/Fm* failed to recover fully in either population, the reduction was minimal (4%) in the southern edge population and was significantly larger (26%) in the northern population (Fig 1; Table 2 and Table A in S1 File). Overall, these results suggest clear variations in the thermal response of PSII between these two edge populations, which reflect differential resilience to thermal stress.

**Table 1:** PERMANOVA of *Fucus vesiculosus Fv/Fm*. *F*. *vesiculosus* from northern and southern distribution edges (Ed: North and South) collected directly after exposure to different temperatures for 3 hours (Te: 10, 18, 24, 28 and 32°C). Significant terms are shown in bold.

Source	df	SS	MS	Pseudo-F	P(perm)
Ed	1	0.10998	0.10998	49.946	**0.001**
Te	4	0.48385	0.12096	54.934	**0.001**
EdxTe	4	0.10246	2.56E-02	11.633	**0.001**
Res	40	8.81E-02	2.20E-03		
Total	49	0.78437			

**Table 2:** PERMANOVA of *Fucus vesiculosus Fv/Fm*. *F*. *vesiculosus* from northern and southern distribution edges (Ed: North and South) subjected to different temperatures for 3 hours (Te: 10, 18, 24, 28 and 32°C) and collected 21 hours after return to control (10°C) temperature. Significant terms are shown in bold, Res = Residual.

Source	df	SS	MS	Pseudo-F	P(perm)
Ed	1	3.28E-02	3.28E-02	22.728	**0.001**
Te	4	0.15281	3.82E-02	26.498	**0.001**
EdxTe	4	9.39E-02	2.35E-02	16.279	**0.001**
Res	40	5.77E-02	1.44E-03		
Total	49	0.33713			

The maximum quantum yield of PSII in *Zostera marina* populations was less severely impacted by HS up to 32°C than in *F*. *vesiculosus* (Figs 1 and 2). The effects of HS at 3 h were not different between populations (Edge; Table 3). The effects of temperature were clearer at 32°C in the northern population, in which inter-individual variation was lower (Fig 2; Table 3, Tables A and B in S1 File). Neither population fully recovered following exposure at 32°C (Fig 2) and PERMANOVA indicated significant differences during recovery due to the main effects temperature and edge, with no interaction (Table 4). The data, therefore, indicate that southern edge populations of *Z*. *marina*, with larger individual response variability, are slightly (but significantly, *p* = 0.045, see Table B in S1 File) less resilient to thermal stress than those at the northern leading edge (Fig 2).

**Table 3:** PERMANOVA of *Zostera marina Fv/Fm*. *Z*. *marina* from northern and southern distribution edges (Ed: North and South) collected directly after exposure to different temperatures for 3 hours (Te: 10, 18, 24, 28 and 32°C). Significant terms are shown in bold, Res = Residual.

Source	df	SS	MS	Pseudo-F	P(perm)
Ed	1	3.36E-05	3.36E-05	7.21E-03	0.929
Te	4	6.51E-02	1.63E-02	3.4875	**0.014**
EdxTe	4	2.31E-03	5.77E-04	0.12374	0.975
Res	40	0.18658	4.66E-03		
Total	49	0.25399			

**Table 4:** PERMANOVA of *Zostera marina Fv/Fm*. *Z*. *marina* from northern and southern distribution edges (Ed: North and South) subjected to different temperatures for 3 hours (Te: 10, 18, 24, 28 and 32°C) and collected 21 hours after return to control (10°C) temperature. Significant terms are shown in bold, Res = Residual.

Source	df	SS	MS	Pseudo-F	P(perm)
Ed	1	2.96E-02	2.96E-02	5.2424	**0.031**
Te	4	5.84E-02	1.46E-02	2.5827	**0.042**
EdxTe	4	2.46E-02	6.14E-03	1.0867	0.380
Res	40	0.22602	5.65E-03		
Total	49	0.33857			

The photosynthetic results were supported by visual tissue inspections: at the highest temperature tissue was discolored at the end of the experiment, whereas at low and medium temperatures no visual changes were observed.

### Gene expression of *Fucus vesiculosus*

**Fig 3:**
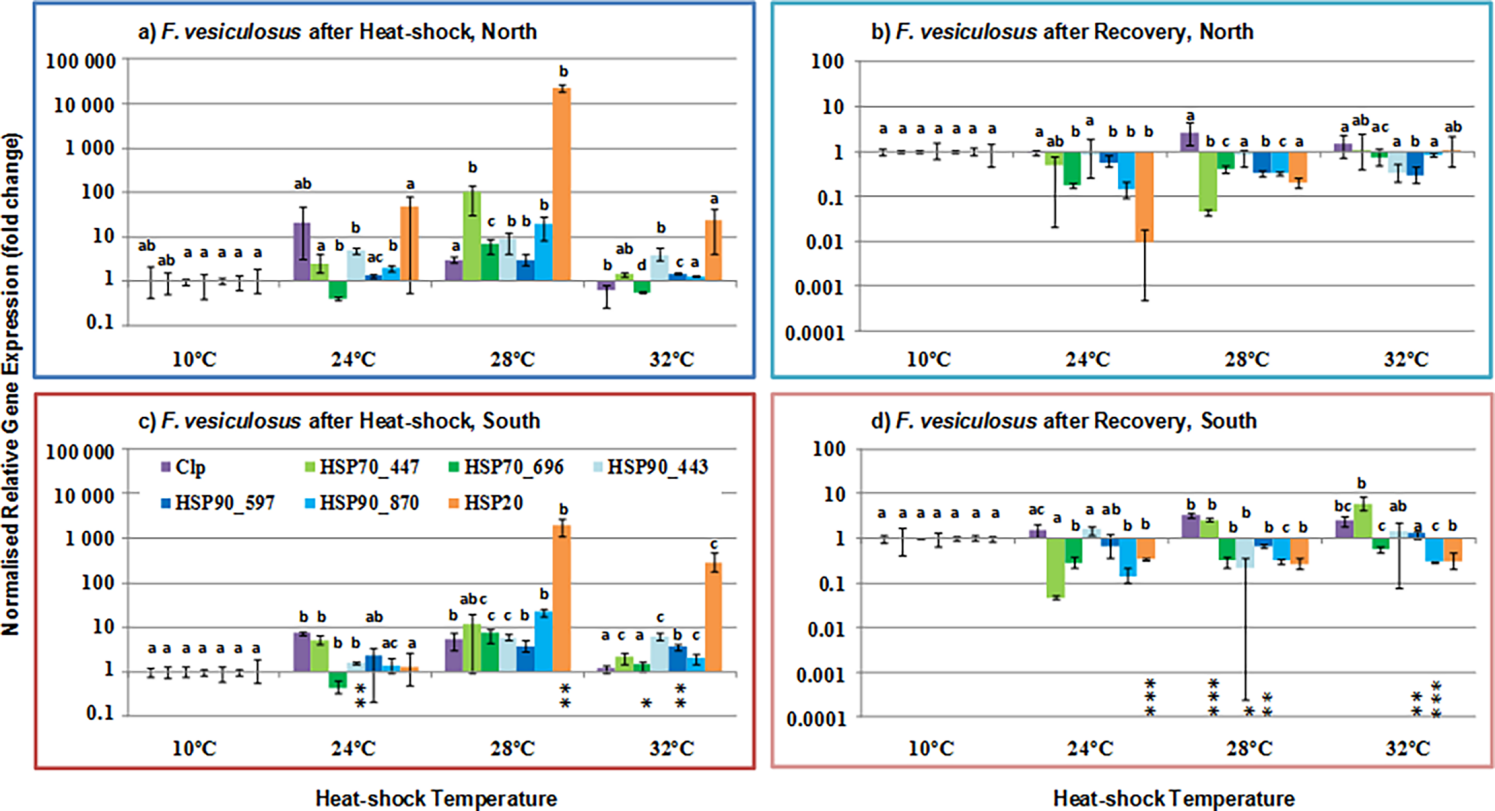
Gene expression temperature response of *Fucus vesiculosus*. Expression of seven heat shock transcripts in response to control (10°C) and elevated seawater temperatures (24, 28 and 32°C), after 3h of HS and after recovery in the alga *Fucus vesiculosus* from the leading (North) and trailing (South) edge of distribution. Relative gene expression values were normalised to geometrical average of three reference genes and to a reference condition (each control at 10°C). Bars show the mean, and error bars display the range of the data (n = 3 biological replicates). Asterisks show significant pair-wise differences between edges (* p<0.05; ** p<0.01; *** p<0.001) and different letters indicate significant pair-wise differences between temperatures (p<0.05).

Despite the diversity of expression patterns among the seven target genes, they all displayed significant expression changes with temperature, as expected for putative HSP transcripts. Immediately after exposure to high temperatures (3 h), most target genes showed increased expression levels (Fig 3A and 3C) and maximum expression occurred at 28°C in both edges (five and six genes for the Northern and Southern edge, respectively, Fig 3, Tables E and G in S1 File). After 3 h HS, significant differences between edges were detected for four genes. Two transcripts (HSP90_443 at 24°C and HSP20 at 28°C) were more abundant in the North edge population, with double and an order of magnitude increase, respectively, while HSP70_696 and HSP90_597 had approximately doubled in expression level in the southern edge after exposure to 32°C (asterisks in Fig 3C, Table E in S1 File). Significant interactions (Ed x Te) during the heat-shock were only detected for HSP70_447, HSP90_443, and HSP20 (Table 5).

**Table 5:** Summary statistical analysis table of *Fucus vesiculosus* gene expression. For each transcript, data from the two sampling times were analysed separately for homogeneity of multivariate dispersions (PERMDISP) and variance (PERMANOVA), for both fixed factors (Edge: North and South; Temperature: 10, 24, 28 and 32°C) with the PERMANOVA module on Primer 6 (Tables C and D in S1 File). Significant *p*-values (<0.05) are shown in bold.

P (perm)		PERMDISP		PERMANOVA		
Time	Gene	Temp	Edge	Edge	Temp	EdxTe
3h HS	Clp_HSP100	**0.003**	0.290	0.675	**0.003**	0.340
3h HS	HSP70_447	**0.001**	**0.001**	**0.010**	**0.001**	**0.005**
3h HS	HSP70_696	**0.002**	0.936	0.463	**0.001**	0.891
3h HS	HSP20	**0.001**	**0.001**	**0.003**	**0.001**	**0.004**
3h HS	HSP90_443	**0.005**	0.681	0.163	**0.001**	**0.035**
3h HS	HSP90_597	**0.025**	**0.047**	**0.022**	**0.005**	0.256
3h HS	HSP90_870	**0.001**	0.822	0.762	**0.001**	0.940
Recovery	Clp_HSP100	0.057	0.642	0.063	**0.003**	0.649
Recovery	HSP70_447	**0.002**	**0.002**	**0.001**	**0.001**	**0.001**
Recovery	HSP70_696	0.283	0.520	0.598	**0.001**	0.444
Recovery	HSP20	0.222	0.312	0.638	**0.009**	0.144
Recovery	HSP90_443	**0.020**	0.123	0.248	0.285	0.105
Recovery	HSP90_597	**0.002**	0.713	**0.001**	**0.011**	**0.005**
Recovery	HSP90_870	**0.001**	0.544	**0.004**	**0.001**	**0.002**

HSP expression peaked at 28°C, likely reflecting a tolerance threshold. In contrast, HSP over-expression was absent after 24 h (Fig 3B and 3D), which may reflect either full recovery (no need for additional HSP production, likely the case below 28°C), or a stress so intense that transcription is still impaired after this period (may be the case after 32°C in the northern edge, when *Fv/Fm* is similarly impacted, Fig 1B).

After recovery, gene expression had mostly returned to control levels (10°C), except for two genes that were still slightly elevated (Clp and HSP70_447 at 28 and 32°C) in the southern population (Fig 3D). Despite similar patterns, significant differentiation between leading and trailing edge populations could be detected in five target genes and at all three HS temperatures (asterisks in Fig 3D). Significant interactions (Ed x Te) after recovery were detected for HSP70_447, HSP90_597 and HSP90_870 (Table 5). Unsurprisingly, most analyses exposed significant heterogeneity of dispersions (Table 5), particularly for temperature, as changes in relative gene expression (fold) often present large inter-replicate variability, resulting from some strong switches (“on-off”) in gene expression, as well as underlying biological variation. As with the *Fv/Fm* measurements, HSP gene expression supports the existence of edge specific differentiation in the induction of a heat shock response. Although statistically supported by only one gene (HSP20), there was a trend towards greater HSP induction in the northern edge population at 28°C, the temperature for peak HS response, while the same population was marginally less able to mount a HS response at 32°C, where *Fv/Fm* was also impacted.

## Discussion

Our results support the differentiation of thermal stress responses between populations located at the leading and trailing edges. Clear population differences observed in the intertidal fucoid alga *Fucus vesiculosus* indicate local adaptation at the southern edge. In contrast, only minor differences were detected in the shallow subtidal seagrass *Zostera marina*. Interestingly, contradicting expectations from the thermal pressures, the data suggest a lower capacity to withstand warmer temperature at the southern edge of *Z*. *marina*. Several hypotheses can be raised to explain these results (discussed below). Both species share similar latitudinal distributions, although their habitats are quite different (intertidal versus subtidal), with the intertidal species commonly exposed to much more variable thermal conditions (in air and water) compared with the subtidal species, which may only infrequently experience short-term changes in seawater temperatures.

The differential resilience to heat-shock of these edge populations raises different hypotheses; one is local adaptation, supported in *F*. *vesiculosus* by the intraspecific differentiation between northern and southern edge populations in physiological and gene expression responses to temperature shifts under common experimental conditions. This is in agreement with previous studies showing that southern and northern groups of *F*. *vesiculosus* form distinct phylogenetic lineages that could support some degree of functional divergence [52,53]. However, despite acclimation to common conditions, there is always the possibility that previous environmental, or parental effects may influence the realised phenotype [54,55]. Nevertheless, our results clearly demonstrated that northern (leading edge) populations of the intertidal species *F*. *vesiculosus* were affected by warming at lower threshold temperatures (beyond 24°C) and showed stronger inhibitory effects at 32°C, while the southern trailing edge seemed well adapted to short-term peaks of water temperature approaching 30°C. The higher thermal resilience, in resistance and recovery, in the south is offset by a greater likelihood of experiencing heat stress events, given the more extreme local temperatures there.

Interestingly, our results suggest that the predominantly subtidal seagrass species *Z*. *marina* may have lower fitness at the southern edge, although the parameters used here (3 h exposure to seawater up to 32°C) did not cause strong physiological impacts on this species. Higher temperatures or longer exposures might improve detection of tolerance differences between edges for this species. A 16 days exposure of northern-edge versus central *Z*. *marina* populations to a temperature range also showed relatively similar temperature optima, although northern-edge populations responded more positively to projected temperature increases than a central Danish population, currently growing at near optimal summer temperatures [41]. A three-week simulated heat wave at 26°C revealed similar transcriptomic profiles between central range (Denmark and USA) and southern range (Italy and USA) populations of *Z*. *marina* during the acute stress, but similarly to our results, population differences increased during recovery [28,33,56]. However, in that study [56] it was the southern population from Italy that seemed more resilient, as expected in case of local adaptation, and a similar result has been observed on a related intertidal species [33]. The subtidal habitat buffers the impact of natural short-term temperature shifts, allowing the persistence of less tolerant species, excluded from higher shore areas. This would lead to the expectation that for the same geographical range, subtidal populations occur in a less selective environment with respect to temperature. In the atidal Baltic sea, *F*. *radicans* was more sensitive to heat-shock than its sister (typically intertidal) species *F*. *vesiculosus* even in common subtidal populations [32]. The higher susceptibility of southern edge populations here found for subtidal *Z*. *marina* has however also been found in intertidal species, namely *F*. *serratus*, which at its southernmost limit (Portugal [7] and Spain [30]) presented lower thermal resilience than central populations, facing greater risks of local extinction [7,30]. The low genetic diversity of these marginal populations was suggested to limit local adaptation [7]. It can be hypothesized that the potentially lower resilience of the southern *Z*. *marina* trailing edge population in this study may result from decreased genetic variability in these small, fragmented and highly clonal populations, as selection is limited by the existing local variability. Although both range edges, Greenland and Portugal, are formed of small meadows with very low diversity [39], currently only the southern edge meadows experience high selective pressure to cope with temperatures above its physiological tolerance.

Selection pressure favouring local adaptation can be counteracted by gene flow with populations from less selective habitats. Local adaptation is therefore expected to be more likely in species with strongly limited dispersal, although with exceptions [57], as is the case of species of *Fucus* and *Zostera*. In the latter, distinct locally adapted populations existed until recently at the southern limit [58]. However, such adaptive traits have not prevented recent local extinctions at many southern edge sites [59], as has also been the case for intertidal fucoid species [60,61]. Current population resilience to increasing temperatures is the outcome of opposing effects: the higher selective pressures would be expected to increase the frequency of the best adapted genotypes. However, the low population genetic diversity at the range edges [39,53] might limit the variation upon which selection can operate, and even result in reduced fitness [7]. The future of edge populations depends on the future (mis)match between local climatic conditions and population resilience, continuously shaped by gene flow and adaptation.

The short-term experimental thermal limits identified here are much higher than realistic levels expected for the near future at the northern edge, suggesting favourable conditions for northwards expansions with global change, in contrast with the recent southern range contractions recorded for these species. Despite lower resilience at the cold leading edge, *Fucus vesiculosus* in the Arctic will likely benefit from increases in temperatures and in ice-free days [62]. Similarly, *Z*. *marina* meadows in Greenland are currently restricted to warmer inner fjord areas and higher temperatures are expected to increase productivity [37]. Northern populations are presumably limited by low temperature and far from becoming impacted by heat-stress, although extreme temperature events, like the 2003 Europe wide heat wave, are expected to increase in frequency and intensity [63]. In this study, both leading edge populations were only impacted at temperatures higher than those predicted for the polar regions, and their range is thus expected to expand with global change. Clearly, the translation of results from *ex situ* short term experiments to *in situ* conditions is complex, but with the contemporary climatic changes [64,65], the role of discrete events, as simulated here, is becoming increasingly clear [66,67]. In addition, the lack of a reference genome and more detailed knowledge of physiological pathways in *Fucus*, precludes us at this time from using the gene expression results beyond their function as biochemical markers of stress.

Although northern leading edge populations face higher warming rates, southern trailing edge populations may be at risk due to higher absolute temperature values, despite having higher thermal resilience. Southern populations have been [59,60] and will likely continue to be undergoing range contractions [53]. This may lead to the loss of highly diverse southern populations [57], thereby likely reducing genetic diversity and the species’ ability to evolve by selection of locally adapted ecotypes. However, unlike *Z*. *marina* (this study) and *F*. *serratus* [7,30], southern populations of *F*. *vesiculosus* may be better adapted to high temperatures than northern populations. Still, such adaptive traits may in the future be adequate for persistence further north but current resilience and adaptive potential may be insufficient to counter further climatic changes at their present southern limit [59,60].

Modern-day species distribution models predict species responses to climate change integrating sub-optimal and lethal physiological responses [68,69]. However, many still typically assume that all populations within a species have uniform constraints. Our results show that although global trends are predictable, more detailed impacts at local scales may be more difficult to predict due to differences in the responses among populations. To predict critical points, it is important to provide temperature response data across temporal scales and consider both the expected temperature shifts and the thermal resilience of the local resident populations.

Given the limited information currently available the physiological implications of the individual gene responses remain speculative. Two HSP70 sequences were annotated further as being cytosol- and chloroplast-targetted. However, multiple members of each HSP family are likely to be present in the *Fucus* genome, as seen with the three HSP90 genes examined, each with distinct (and mostly unknown) sub-cellular localization, expression and protein interaction patterns.

It is beyond the scope of this paper to detail the current knowledge on the heat-shock response and the function of heat-shock proteins (HSPs). Multiple review papers deal with these very conserved families, exploring HSP functions from humans to plants and yeast (e.g., [70,71,72,73]). The roles of HSPs extend far beyond protection from elevated temperatures and are still not fully understood, for example, the role of HSP90 in buffering genetic variation, and modifying the phenotypic expression of genetic variants [74, 75]. Knowledge of HSP functions in the brown algal lineage is still lacking, although intertidal *Fucus vesiculosus* may have more HSP genes than *Ectocarpus siliculosus*, considering the small genome of the brown alga model organism.

The small HSPs are known to be rapidly and strongly induced as large amounts are required to bind to and stabilize a variety of damaged, unfolded and aggregated proteins [76, 77]. HSP20 was expressed at very low levels at 10°C and dramatically increased at high temperatures, as expected and previously described [7, 32, 45]. The absence of induction at 24°C likely reflects lower stress in the southern population (fewer misfolded proteins present); lower transcript levels in the northern population at 32°C is likely because thermal limits were exceeded. Other HSPs must then actively repair or remove these damaged proteins. ClpB/HSP100 works with Hsp70 to refold protein aggregates [76, 78, 79]. Unlike HSP20s, their activity requires ATP, but these HSPs are recycled for a new refolding cycle. Non-proteolytic ClpB (Clp_7H01) is increased at 24 and 28°C in the southern population. Lower expression at 28°C in the northern population might reflect the situation at 32°C, when the cells can no longer sustain a heat shock response, although this is not supported by the other genes.

DnaK (HSP70_447) is chloroplast encoded and its increase at 28°C should reflect an effort to sustain photosynthetic activity as it starts to be heat inactivated [80]. HSP90 (like HSP70) is essential and abundant in unstressed cells [70, 81, 82]. HSP90 (with Heat Shock Factor and other chaperones) regulates the heat shock response and its own transcription [83] so it should not be strongly induced. HSP90 genes can be constitutively expressed with marginal induction under heat stress. These small changes can help regulate the cell to a new steady-state level of HSPs, thus allowing acclimation. After return to control temperatures the cells will gradually return to normal metabolism, once damaged proteins are repaired or removed. The time it takes for HSPs either to return to control levels or stabilise in a new equilibrium reflects the recovery time and will depend on the severity of the stress and on the level and activity of HSPs.

By contrasting two widespread ecosystem-structuring macrophyte species with similar latitudinal ranges, our study shows that leading and trailing edge populations can have different responses to thermal stress. Moreover, the thermal responses supported the hypothesis that fitness and resilience to warming can differ between cold and warm range edges of widespread ecosystem-structuring, coastal macrophytes. However, the apparent species-specific nature of response, despite apparent similar climatic affinities, makes it difficult to discern overarching patterns at this point. While the causes underlying these distinct patterns and predictions between the two species models are not clear at this time, we raise the hypothesis that low fitness-related genetic variability might be restricting evolvability (*sensu* Pearson *et al*. [7]).

Besides suggesting possible susceptibility to climatic changes, the population differences reported here have practical consequences. Activities leading to admixture of differentiated populations might result in less fit offspring due to outbreeding depression, reduced fitness in offspring from crosses between divergent genotypes, a major concern for conservation management. Habitat restoration practices should take into account the possible occurrence of locally adapted genotypes and consider these a conservation priority as well as the best source for population restoration at similar habitats where populations have become extinct.

## Supporting information

S1 File Table A. Pairwise tests for *Fv/Fm* data. Table B. Pairwise tests for the significant factors (Edge or Temperature) in *Z*. *marina Fv/Fm* data. Table C. PERMANOVA results for normalised relative expression of seven genes after the 3h HS (n = 3). Table D. PERMANOVA results for normalised relative expression of seven genes during Recovery (n = 3). Table E. Pair-wise tests for gene expression data. Table F. *Fv/Fm* raw data. Table G. Relative gene expression data of the technical duplicates normalised to the geometric average of three reference genes.
(DOCX)
