# Supplementary material for: Differentiation in fitness-related traits in response to elevated temperatures between leading and trailing edge populations of marine macrophytes
Source: PLoS One. 2018 Sep 13;13(9):e0203666. doi: 10.1371/journal.pone.0203666 (PMC6136734; doi:10.1371/journal.pone.0203666)
Supplement: S1 File — Table A. Pairwise tests for Fv/Fm data. Table B. Pairwise tests for the significant factors (Edge or Temperature) in Z. marina Fv/Fm data. Table C. PERMANOVA results for normalised relative expression of seven genes after the 3h HS (n = 3). Table D. PERMANOVA results for normalised relative expression of seven genes during Recovery (n = 3). Table E. Pair-wise tests for gene expression data. Table F. Fv/Fm raw data. Table G. Relative gene expression data of the technical duplicates normalised to the geometric average of three reference genes. (DOCX) [file pone.0203666.s001.docx]

**Differentiation in fitness-related traits in response to elevated temperatures between leading and trailing edge populations of marine macrophytes**

Aschwin H. Engelen^*^, Catarina Mota^*^, Ester A. Serrao, Márcio Coelho, Núria Marbà, Dorte Krause-Jensen, Gareth A. Pearson (^*^**Authors with equal contribution**)

**Supplementary Information**

Supplementary Table A – Pairwise tests for Fv/Fm data. *Fv/Fm* values (n=5), normalised to each 10ºC control (raw data on Table F), were used to build a resemblance matrix of Euclidean distances between samples. Analysis were made with 999 unrestricted permutations of raw data, fixed effects sum to zero for mixed terms and Type III (partial) sums of squares, in the PERMANOVA module of Primer v6 software.

| **Sample group** | | **Pair-wise tests for factors (Edge or Temp) within "Ed x Te"** | | | | | |
| --- | --- | --- | --- | --- | --- | --- | --- |
| **Species** | **Time** | **Factor** | **Groups** | **t** | **P(perm)** | **Unique perms** | **P(MC)** |
| Fves | 3h HS | North | 10, 18 | 2.5411 | 0.045 | 63 | **0.036** |
| Fves | 3h HS | North | 10, 24 | 2.4578 | 0.055 | 88 | **0.035** |
| Fves | 3h HS | North | 10, 28 | 4.9112 | 0.010 | 106 | **0.002** |
| Fves | 3h HS | North | 10, 32 | 9.9277 | 0.011 | 126 | **0.001** |
| Fves | 3h HS | North | 18, 24 | 0.59456 | 0.562 | 76 | 0.584 |
| Fves | 3h HS | North | 18, 28 | 3.0485 | 0.021 | 97 | **0.023** |
| Fves | 3h HS | North | 18, 32 | 8.6943 | 0.016 | 120 | **0.001** |
| Fves | 3h HS | North | 24, 28 | 2.1559 | 0.063 | 89 | 0.082 |
| Fves | 3h HS | North | 24, 32 | 7.8214 | 0.007 | 116 | **0.001** |
| Fves | 3h HS | North | 28, 32 | 6.1671 | 0.006 | 112 | **0.003** |
| Fves | 3h HS | South | 10, 18 | 0.72111 | 0.568 | 58 | 0.487 |
| Fves | 3h HS | South | 10, 24 | 1.2647 | 0.255 | 33 | 0.231 |
| Fves | 3h HS | South | 10, 28 | 0.26092 | 0.845 | 80 | 0.810 |
| Fves | 3h HS | South | 10, 32 | 4.7048 | 0.014 | 103 | **0.003** |
| Fves | 3h HS | South | 18, 24 | 1.2935 | 0.248 | 58 | 0.209 |
| Fves | 3h HS | South | 18, 28 | 0.59738 | 0.586 | 90 | 0.596 |
| Fves | 3h HS | South | 18, 32 | 4.6293 | 0.004 | 109 | **0.002** |
| Fves | 3h HS | South | 24, 28 | 5.39E-02 | 0.978 | 70 | 0.952 |
| Fves | 3h HS | South | 24, 32 | 4.4366 | 0.007 | 85 | **0.002** |
| Fves | 3h HS | South | 28, 32 | 3.3888 | 0.025 | 110 | **0.012** |
| Fves | 3h HS | 10 | North, South | 2.01E-02 | 1.000 | 44 | 0.986 |
| Fves | 3h HS | 18 | North, South | 2.6574 | 0.028 | 78 | **0.023** |
| Fves | 3h HS | 24 | North, South | 2.2297 | 0.082 | 53 | 0.062 |
| Fves | 3h HS | 28 | North, South | 3.2902 | 0.025 | 96 | **0.014** |
| Fves | 3h HS | 32 | North, South | 5.3607 | 0.009 | 86 | **0.004** |
| Fves | Recovery | North | 10, 18 | 1.7555 | 0.112 | 68 | 0.105 |
| Fves | Recovery | North | 10, 24 | 1.7774 | 0.114 | 67 | 0.125 |
| Fves | Recovery | North | 10, 28 | 1.717 | 0.107 | 63 | 0.120 |
| Fves | Recovery | North | 10, 32 | 5.637 | 0.009 | 109 | **0.002** |
| Fves | Recovery | North | 18, 24 | 0.10844 | 0.942 | 59 | 0.904 |
| Fves | Recovery | North | 18, 28 | 0.39684 | 0.709 | 61 | 0.717 |
| Fves | Recovery | North | 18, 32 | 5.0394 | 0.007 | 115 | **0.001** |
| Fves | Recovery | North | 24, 28 | 0.30883 | 0.761 | 57 | 0.761 |
| Fves | Recovery | North | 24, 32 | 5.1164 | 0.011 | 111 | **0.001** |
| Fves | Recovery | North | 28, 32 | 5.2517 | 0.012 | 116 | **0.002** |
| Fves | Recovery | South | 10, 18 | 2.6735 | 0.035 | 69 | **0.027** |
| Fves | Recovery | South | 10, 24 | 0.55459 | 0.619 | 45 | 0.582 |
| Fves | Recovery | South | 10, 28 | 0.51471 | 0.617 | 56 | 0.616 |
| Fves | Recovery | South | 10, 32 | 4.5903 | 0.010 | 67 | **0.002** |
| Fves | Recovery | South | 18, 24 | 2.0797 | 0.070 | 52 | 0.082 |
| Fves | Recovery | South | 18, 28 | 1.7292 | 0.131 | 67 | 0.116 |
| Fves | Recovery | South | 18, 32 | 0.30311 | 0.786 | 60 | 0.756 |
| Fves | Recovery | South | 24, 28 | 8.33E-02 | 0.968 | 51 | 0.933 |
| Fves | Recovery | South | 24, 32 | 3.3087 | 0.029 | 50 | **0.011** |
| Fves | Recovery | South | 28, 32 | 2.4824 | 0.074 | 50 | **0.039** |
| Fves | Recovery | 10 | North, South | 1.40E-08 | 1.000 | 64 | 1.000 |
| Fves | Recovery | 18 | North, South | 0.40953 | 0.721 | 72 | 0.696 |
| Fves | Recovery | 24 | North, South | 1.7633 | 0.129 | 33 | 0.123 |
| Fves | Recovery | 28 | North, South | 1.279 | 0.260 | 65 | 0.254 |
| Fves | Recovery | 32 | North, South | 4.8986 | 0.007 | 116 | **0.002** |
| Zmar | 3h HS | North | 10, 18 | 1.5454 | 0.143 | 45 | 0.156 |
| Zmar | 3h HS | North | 10, 24 | 1.5678 | 0.127 | 80 | 0.177 |
| Zmar | 3h HS | North | 10, 28 | 1.36E-08 | 1.000 | 71 | 1.000 |
| Zmar | 3h HS | North | 10, 32 | 3.2487 | 0.020 | 90 | **0.012** |
| Zmar | 3h HS | North | 18, 24 | 2.7812 | 0.047 | 69 | **0.020** |
| Zmar | 3h HS | North | 18, 28 | 1.3626 | 0.249 | 53 | 0.238 |
| Zmar | 3h HS | North | 18, 32 | 4.7995 | 0.005 | 97 | **0.003** |
| Zmar | 3h HS | North | 24, 28 | 1.4912 | 0.160 | 85 | 0.163 |
| Zmar | 3h HS | North | 24, 32 | 1.2814 | 0.203 | 88 | 0.224 |
| Zmar | 3h HS | North | 28, 32 | 3.0693 | 0.017 | 91 | **0.020** |
| Zmar | 3h HS | South | 10, 18 | 0.3868 | 0.709 | 100 | 0.698 |
| Zmar | 3h HS | South | 10, 24 | 0.86001 | 0.506 | 78 | 0.401 |
| Zmar | 3h HS | South | 10, 28 | 0.66904 | 0.487 | 94 | 0.531 |
| Zmar | 3h HS | South | 10, 32 | 1.2274 | 0.232 | 122 | 0.246 |
| Zmar | 3h HS | South | 18, 24 | 1.1303 | 0.299 | 102 | 0.280 |
| Zmar | 3h HS | South | 18, 28 | 0.22233 | 0.865 | 76 | 0.822 |
| Zmar | 3h HS | South | 18, 32 | 1.458 | 0.170 | 107 | 0.206 |
| Zmar | 3h HS | South | 24, 28 | 1.351 | 0.231 | 94 | 0.201 |
| Zmar | 3h HS | South | 24, 32 | 0.42132 | 0.634 | 115 | 0.701 |
| Zmar | 3h HS | South | 28, 32 | 1.6488 | 0.126 | 63 | 0.141 |
| Zmar | 3h HS | 10 | North, South | 6.47E-03 | 1.000 | 85 | 0.996 |
| Zmar | 3h HS | 18 | North, South | 0.19055 | 0.922 | 79 | 0.857 |
| Zmar | 3h HS | 24 | North, South | 0.25029 | 0.896 | 107 | 0.816 |
| Zmar | 3h HS | 28 | North, South | 0.9526 | 0.368 | 81 | 0.375 |
| Zmar | 3h HS | 32 | North, South | 0.20477 | 0.913 | 103 | 0.865 |
| Zmar | Recovery | North | 10, 18 | 0.16662 | 0.899 | 45 | 0.877 |
| Zmar | Recovery | North | 10, 24 | 0.5108 | 0.632 | 98 | 0.612 |
| Zmar | Recovery | North | 10, 28 | 0.47517 | 0.663 | 85 | 0.665 |
| Zmar | Recovery | North | 10, 32 | 2.36 | 0.044 | 112 | **0.048** |
| Zmar | Recovery | North | 18, 24 | 0.57717 | 0.613 | 65 | 0.573 |
| Zmar | Recovery | North | 18, 28 | 0.49244 | 0.647 | 79 | 0.650 |
| Zmar | Recovery | North | 18, 32 | 2.6306 | 0.021 | 91 | **0.025** |
| Zmar | Recovery | North | 24, 28 | 2.13E-02 | 0.996 | 75 | 0.984 |
| Zmar | Recovery | North | 24, 32 | 2.384 | 0.016 | 111 | **0.048** |
| Zmar | Recovery | North | 28, 32 | 2.3336 | 0.023 | 96 | **0.048** |
| Zmar | Recovery | South | 10, 18 | 3.7225 | 0.019 | 85 | **0.006** |
| Zmar | Recovery | South | 10, 24 | 1.5857 | 0.090 | 94 | 0.174 |
| Zmar | Recovery | South | 10, 28 | 1.8964 | 0.069 | 90 | 0.079 |
| Zmar | Recovery | South | 10, 32 | 3.3572 | 0.008 | 97 | **0.014** |
| Zmar | Recovery | South | 18, 24 | 0.81901 | 0.621 | 110 | 0.451 |
| Zmar | Recovery | South | 18, 28 | 0.95145 | 0.434 | 110 | 0.413 |
| Zmar | Recovery | South | 18, 32 | 1.5464 | 0.105 | 91 | 0.172 |
| Zmar | Recovery | South | 24, 28 | 4.05E-02 | 0.962 | 99 | 0.973 |
| Zmar | Recovery | South | 24, 32 | 0.1859 | 0.880 | 106 | 0.847 |
| Zmar | Recovery | South | 28, 32 | 0.16532 | 0.881 | 106 | 0.865 |
| Zmar | Recovery | 10 | North, South | 7.71E-02 | 0.963 | 88 | 0.947 |
| Zmar | Recovery | 18 | North, South | 3.418 | 0.023 | 56 | **0.010** |
| Zmar | Recovery | 24 | North, South | 1.4088 | 0.201 | 106 | 0.213 |
| Zmar | Recovery | 28 | North, South | 1.6651 | 0.157 | 109 | 0.135 |
| Zmar | Recovery | 32 | North, South | 0.11597 | 0.834 | 74 | 0.908 |

Supplementary Table B – Pairwise tests for the significant factors (Edge or Temperature) in *Z. marina Fv/Fm* data. Analyses were made as previously described using the PERMANOVA module of Primer v6 software (data from Table F, normalised to each control condition).

| **Time** | **Factor** | **Groups** | **t** | **P(perm)** | **Unique perms** | **P(MC)** |
| --- | --- | --- | --- | --- | --- | --- |
| 3h HS | Temp | 10, 18 | 0.86769 | 0.389 | 990 | 0.434 |
| 3h HS | Temp | 10, 24 | 1.3783 | 0.182 | 992 | 0.181 |
| 3h HS | Temp | 10, 28 | 0.59086 | 0.579 | 993 | 0.590 |
| 3h HS | Temp | **10, 32** | **2.1528** | **0.034** | **998** | **0.046** |
| 3h HS | Temp | 18, 24 | 2.0009 | 0.052 | 991 | 0.072 |
| 3h HS | Temp | 18, 28 | 0.35382 | 0.704 | 983 | 0.729 |
| 3h HS | Temp | **18, 32** | **2.7016** | **0.008** | **998** | **0.018** |
| 3h HS | Temp | 24, 28 | 1.8409 | 0.067 | 996 | 0.096 |
| 3h HS | Temp | 24, 32 | 0.80436 | 0.469 | 994 | 0.455 |
| 3h HS | Temp | **28, 32** | **2.5737** | **0.013** | **995** | **0.013** |
| **Time** | **Factor** | **Groups** | **t** | **P(perm)** | **Unique perms** | **P(MC)** |
| Recovery | Temp | 10, 18 | 1.8318 | 0.094 | 992 | 0.082 |
| Recovery | Temp | 10, 24 | 1.6602 | 0.069 | 992 | 0.129 |
| Recovery | Temp | 10, 28 | 1.8989 | 0.075 | 992 | 0.078 |
| Recovery | Temp | 10, 32 | 3.8088 | **0.003** | 994 | 0.006 |
| Recovery | Temp | 18, 24 | 0.92959 | 0.448 | 996 | 0.372 |
| Recovery | Temp | 18, 28 | 1.0562 | 0.363 | 990 | 0.319 |
| Recovery | Temp | 18, 32 | 3.0264 | **0.002** | 994 | 0.005 |
| Recovery | Temp | 24, 28 | 4.39E-02 | 0.967 | 996 | 0.975 |
| Recovery | Temp | 24, 32 | 0.9165 | 0.417 | 996 | 0.388 |
| Recovery | Temp | 28, 32 | 1.1021 | 0.309 | 998 | 0.288 |
| **Time** | **Factor** | **Groups** | **t** | **P(perm)** | **Unique perms** | **P(MC)** |
| Recovery | Edge | North, South | 2.1976 | **0.045** | 993 | 0.020 |

Supplementary Table C – PERMANOVA results for normalised relative expression of seven genes after the 3h HS (n=3). Analyses were made as previously described using the PERMANOVA module of Primer v6 software (data from Table G, normalised to each control condition).

| PERMANOVA table of results for Clp_HSP100 after 3h of HS | | | | | |
| --- | --- | --- | --- | --- | --- |
| Source | df | SS | MS | Pseudo-F | P(perm) |
| Ed | 1 | 42.971 | 42.971 | 0.59527 | 0.675 |
| **Te** | **3** | **731.65** | **243.88** | **3.3785** | **0.003** |
| EdxTe | 3 | 250.43 | 83.478 | 1.1564 | 0.340 |
| Res | 16 | 1155 | 72.187 |  |  |
| Total | 23 | 2180.1 |  |  |  |
|  |  |  |  |  |  |
| PERMANOVA table of results for HSP70_447 after 3h of HS | | | | | |
| Source | df | SS | MS | Pseudo-F | P(perm) |
| **Ed** | **1** | **3055.8** | **3055.8** | **5.6243** | **0.010** |
| **Te** | **3** | **14296** | **4765.3** | **8.7709** | **0.001** |
| **EdxTe** | **3** | **10082** | **3360.6** | **6.1854** | **0.005** |
| Res | 16 | 8693 | 543.31 |  |  |
| Total | 23 | 36127 |  |  |  |
|  |  |  |  |  |  |
| PERMANOVA table of results for HSP70_696 after 3h of HS | | | | | |
| Source | df | SS | MS | Pseudo-F | P(perm) |
| Ed | 1 | 0.80396 | 0.80396 | 0.54679 | 0.463 |
| **Te** | **3** | **176.9** | **58.967** | **40.105** | **0.001** |
| EdxTe | 3 | 0.85273 | 0.28424 | 0.19332 | 0.891 |
| Res | 16 | 23.525 | 1.4703 |  |  |
| Total | 23 | 202.08 |  |  |  |
|  |  |  |  |  |  |
| PERMANOVA table of results for HSP90_443 after 3h of HS | | | | | |
| Source | df | SS | MS | Pseudo-F | P(perm) |
| Ed | 1 | 6.8686 | 6.8686 | 2.1769 | 0.163 |
| **Te** | **3** | **132.28** | **44.094** | **13.975** | **0.001** |
| **EdxTe** | **3** | **32.719** | **10.906** | **3.4566** | **0.035** |
| Res | 16 | 50.484 | 3.1552 |  |  |
| Total | 23 | 222.35 |  |  |  |
|  |  |  |  |  |  |
| PERMANOVA table of results for HSP90_597 after 3h of HS | | | | | |
| Source | df | SS | MS | Pseudo-F | P(perm) |
| **Ed** | **1** | **6.0118** | **6.0118** | **7.2001** | **0.022** |
| **Te** | **3** | **18.299** | **6.0995** | **7.3051** | **0.005** |
| EdxTe | 3 | 3.8213 | 1.2738 | 1.5255 | 0.256 |
| Res | 16 | 13.359 | 0.83497 |  |  |
| Total | 23 | 41.491 |  |  |  |
|  |  |  |  |  |  |
| PERMANOVA table of results for HSP90_870 after 3h of HS | | | | | |
| Source | df | SS | MS | Pseudo-F | P(perm) |
| Ed | 1 | 2.0353 | 2.0353 | 0.12948 | 0.762 |
| **Te** | **3** | **1719.5** | **573.17** | **36.462** | **0.001** |
| EdxTe | 3 | 6.4369 | 2.1456 | 0.13649 | 0.940 |
| Res | 16 | 251.51 | 15.72 |  |  |
| Total | 23 | 1979.5 |  |  |  |
|  |  |  |  |  |  |
| PERMANOVA table of results for HSP20 after 3h of HS | | | | | |
| Source | df | SS | MS | Pseudo-F | P(perm) |
| **Ed** | **1** | **1.60E+08** | **1.60E+08** | **59.095** | **0.003** |
| **Te** | **3** | **6.84E+08** | **2.28E+08** | **84.251** | **0.001** |
| **EdxTe** | **3** | **4.93E+08** | **1.64E+08** | **60.734** | **0.004** |
| Res | 16 | 4.33E+07 | 2.71E+06 |  |  |
| Total | 23 | 1.38E+09 |  |  |  |
|  |  |  |  |  |  |

Supplementary Table D – PERMANOVA results for normalised relative expression of seven genes during Recovery (n=3). Analyses were made as previously described using the PERMANOVA module of Primer v6 software (data from Table G, normalised to each control condition).

| PERMANOVA table of results for Clp_HSP100 after Recovery | | | | | |
| --- | --- | --- | --- | --- | --- |
| Source | df | SS | MS | Pseudo-F | P(perm) |
| Ed | 1 | 2.0867 | 2.0867 | 4.18 | 0.063 |
| **Te** | **3** | **15.495** | **5.1649** | **10.346** | **0.003** |
| EdxTe | 3 | 0.9116 | 0.30387 | 0.60869 | 0.649 |
| Res | 16 | 7.9875 | 0.49922 |  |  |
| Total | 23 | 26.48 |  |  |  |
|  |  |  |  |  |  |
| PERMANOVA table of results for HSP70_447 after Recovery | | | | | |
| Source | df | SS | MS | Pseudo-F | P(perm) |
| **Ed** | **1** | **16.975** | **16.975** | **15.512** | **0.001** |
| **Te** | **3** | **33.944** | **11.315** | **10.34** | **0.001** |
| **EdxTe** | **3** | **25.896** | **8.6319** | **7.8881** | **0.001** |
| Res | 16 | 17.509 | 1.0943 |  |  |
| Total | 23 | 94.324 |  |  |  |
|  |  |  |  |  |  |
| PERMANOVA table of results for HSP70_696 after Recovery | | | | | |
| Source | df | SS | MS | Pseudo-F | P(perm) |
| Ed | 1 | 5.73E-03 | 5.73E-03 | 0.2711 | 0.598 |
| **Te** | **3** | **2.0761** | **0.69205** | **32.763** | **0.001** |
| EdxTe | 3 | 5.80E-02 | 1.93E-02 | 0.91508 | 0.444 |
| Res | 16 | 0.33797 | 2.11E-02 |  |  |
| Total | 23 | 2.4778 |  |  |  |
|  |  |  |  |  |  |
| PERMANOVA table of results for HSP90_443 after Recovery | | | | | |
| Source | df | SS | MS | Pseudo-F | P(perm) |
| Ed | 1 | 6.28E-01 | 6.28E-01 | 1.6466 | 0.248 |
| Te | 3 | 1.5755 | 0.52515 | 1.378 | 0.285 |
| EdxTe | 3 | 2.7505 | 0.91684 | 2.4057 | 0.105 |
| Res | 16 | 6.0978 | 0.38111 |  |  |
| Total | 23 | 11.051 |  |  |  |
|  |  |  |  |  |  |
| PERMANOVA table of results for HSP90_597 after Recovery | | | | | |
| Source | df | SS | MS | Pseudo-F | P(perm) |
| **Ed** | **1** | **7.66E-01** | **7.66E-01** | **15.559** | **0.001** |
| **Te** | **3** | **0.85157** | **0.28386** | **5.7625** | **0.011** |
| **EdxTe** | **3** | **0.93168** | **0.31056** | **6.3045** | **0.005** |
| Res | 16 | 0.78816 | 4.93E-02 |  |  |
| Total | 23 | 3.3378 |  |  |  |
|  |  |  |  |  |  |
| PERMANOVA table of results for HSP90_870 after Recovery | | | | | |
| Source | df | SS | MS | Pseudo-F | P(perm) |
| **Ed** | **1** | **1.23E-01** | **1.23E-01** | **12.778** | **0.004** |
| **Te** | **3** | **2.474** | **0.82465** | **85.75** | **0.001** |
| **EdxTe** | **3** | **0.31844** | **0.10615** | **11.038** | **0.002** |
| Res | 16 | 0.15387 | 9.62E-03 |  |  |
| Total | 23 | 3.0692 |  |  |  |
|  |  |  |  |  |  |
| PERMANOVA table of results for HSP20 after Recovery | | | | | |
| Source | df | SS | MS | Pseudo-F | P(perm) |
| Ed | 1 | 4.74E-02 | 4.74E-02 | 0.28729 | 0.638 |
| **Te** | **3** | **2.7503** | **0.91676** | **5.5572** | **0.009** |
| EdxTe | 3 | 1.005 | 0.33499 | 2.0306 | 0.144 |
| Res | 16 | 2.6395 | 0.16497 |  |  |
| Total | 23 | 6.4421 |  |  |  |

Supplementary Table E – Pair-wise tests for gene expression data. Analyses were made as previously described using the PERMANOVA module of Primer v6 software (data from Table G, normalised to each control condition).

| **Sample group** | | **Pair-wise tests for factors (Edge or Temp) within "Ed x Te"** | | | | | | |
| --- | --- | --- | --- | --- | --- | --- | --- | --- |
| **Gene** | **Time** | **Factor** | **Groups** | **t** | **P(perm)** | **Unique perms** | **P(MC)** | |
| Clp_ HSP100 | 3h HS | 10 | South, North | 7.42E-09 | 1 | 10 | 1 |  |
| Clp_ HSP100 | 3h HS | 24 | South, North | 0.99772 | 0.486 | 10 | 0.341 |  |
| Clp_ HSP100 | 3h HS | 28 | South, North | 1.7377 | 0.197 | 10 | 0.164 |  |
| Clp_ HSP100 | 3h HS | 32 | South, North | 2.8503 | 0.121 | 10 | 0.053 |  |
| Clp_ HSP100 | 3h HS | South | 10, 24 | 11.7 | 0.109 | 10 | **0.001** |  |
| Clp_ HSP100 | 3h HS | South | 10, 28 | 3.2159 | 0.111 | 10 | **0.034** |  |
| Clp_ HSP100 | 3h HS | South | 10, 32 | 1.2984 | 0.320 | 10 | 0.284 |  |
| Clp_ HSP100 | 3h HS | South | 24, 28 | 1.4518 | 0.205 | 10 | 0.226 |  |
| Clp_ HSP100 | 3h HS | South | 24, 32 | 11.281 | 0.106 | 10 | **0.002** |  |
| Clp_ HSP100 | 3h HS | South | 28, 32 | 3.0505 | 0.094 | 10 | **0.049** |  |
| Clp_ HSP100 | 3h HS | North | 10, 24 | 1.4717 | 0.098 | 10 | 0.201 |  |
| Clp_ HSP100 | 3h HS | North | 10, 28 | 3.0151 | 0.096 | 10 | 0.055 |  |
| Clp_ HSP100 | 3h HS | North | 10, 32 | 0.65894 | 0.827 | 10 | 0.538 |  |
| Clp_ HSP100 | 3h HS | North | 24, 28 | 1.3298 | 0.209 | 10 | 0.253 |  |
| Clp_ HSP100 | 3h HS | North | 24, 32 | 1.5013 | 0.094 | 10 | 0.212 |  |
| Clp_ HSP100 | 3h HS | North | 28, 32 | 6.3952 | 0.095 | 10 | **0.005** |  |
| HSP70_447 | 3h HS | 10 | South, North | Negative |  |  |  |  |
| HSP70_447 | 3h HS | 24 | South, North | 2.3999 | 0.113 | 10 | 0.074 |  |
| HSP70_447 | 3h HS | 28 | South, North | 2.4589 | 0.105 | 10 | 0.091 |  |
| HSP70_447 | 3h HS | 32 | South, North | 1.9111 | 0.296 | 10 | 0.134 |  |
| HSP70_447 | 3h HS | South | 10, 24 | 5.1876 | 0.098 | 10 | **0.001** |  |
| HSP70_447 | 3h HS | South | 10, 28 | 1.899 | 0.311 | 10 | 0.119 |  |
| HSP70_447 | 3h HS | South | 10, 32 | 2.9095 | 0.096 | 10 | **0.034** |  |
| HSP70_447 | 3h HS | South | 24, 28 | 1.176 | 0.394 | 10 | 0.286 |  |
| HSP70_447 | 3h HS | South | 24, 32 | 3.4504 | 0.104 | 10 | **0.033** |  |
| HSP70_447 | 3h HS | South | 28, 32 | 1.6926 | 0.439 | 10 | 0.164 |  |
| HSP70_447 | 3h HS | North | 10, 24 | 1.7364 | 0.183 | 10 | 0.159 |  |
| HSP70_447 | 3h HS | North | 10, 28 | 2.7713 | 0.087 | 10 | 0.054 |  |
| HSP70_447 | 3h HS | North | 10, 32 | 1.4821 | 0.308 | 10 | 0.207 |  |
| HSP70_447 | 3h HS | North | 24, 28 | 2.7326 | 0.086 | 10 | **0.038** |  |
| HSP70_447 | 3h HS | North | 24, 32 | 1.2592 | 0.178 | 10 | 0.282 |  |
| HSP70_447 | 3h HS | North | 28, 32 | 2.7591 | 0.105 | 10 | 0.054 |  |
| HSP70_696 | 3h HS | 10 | South, North | 1.91E-05 | 1 | 10 | 1 |  |
| HSP70_696 | 3h HS | 24 | South, North | 0.43391 | 1 | 10 | 0.689 |  |
| HSP70_696 | 3h HS | 28 | South, North | 0.25663 | 0.621 | 10 | 0.797 |  |
| HSP70_696 | 3h HS | 32 | South, North | 3.677 | 0.115 | 10 | **0.016** |  |
| HSP70_696 | 3h HS | South | 10, 24 | 3.1917 | 0.104 | 10 | **0.037** |  |
| HSP70_696 | 3h HS | South | 10, 28 | 4.5525 | 0.09 | 10 | **0.007** |  |
| HSP70_696 | 3h HS | South | 10, 32 | 1.6798 | 0.207 | 10 | 0.174 |  |
| HSP70_696 | 3h HS | South | 24, 28 | 4.9666 | 0.105 | 10 | **0.010** |  |
| HSP70_696 | 3h HS | South | 24, 32 | 3.8999 | 0.093 | 10 | **0.020** |  |
| HSP70_696 | 3h HS | South | 28, 32 | 4.1566 | 0.096 | 10 | **0.019** |  |
| HSP70_696 | 3h HS | North | 10, 24 | 6.1299 | 0.095 | 10 | **0.009** |  |
| HSP70_696 | 3h HS | North | 10, 28 | 4.19 | 0.101 | 10 | **0.011** |  |
| HSP70_696 | 3h HS | North | 10, 32 | 4.5528 | 0.101 | 10 | **0.013** |  |
| HSP70_696 | 3h HS | North | 24, 28 | 4.6257 | 0.095 | 10 | **0.009** |  |
| HSP70_696 | 3h HS | North | 24, 32 | 6.3223 | 0.096 | 10 | **0.004** |  |
| HSP70_696 | 3h HS | North | 28, 32 | 4.5136 | 0.122 | 10 | **0.014** |  |
| HSP20-2 | 3h HS | 10 | South, North | 1.04E-05 | 1 | 5 | 1 |  |
| HSP20-2 | 3h HS | 24 | South, North | 1.932 | 0.289 | 10 | 0.130 |  |
| HSP20-2 | 3h HS | 28 | South, North | 7.7713 | 0.094 | 10 | **0.004** |  |
| HSP20-2 | 3h HS | 32 | South, North | 2.7726 | 0.096 | 10 | 0.058 |  |
| HSP20-2 | 3h HS | South | 10, 24 | 0.40154 | 0.694 | 7 | 0.714 |  |
| HSP20-2 | 3h HS | South | 10, 28 | 4.1952 | 0.095 | 7 | **0.011** |  |
| HSP20-2 | 3h HS | South | 10, 32 | 3.0362 | 0.112 | 7 | **0.050** |  |
| HSP20-2 | 3h HS | South | 24, 28 | 4.1945 | 0.103 | 10 | **0.013** |  |
| HSP20-2 | 3h HS | South | 24, 32 | 3.0326 | 0.101 | 10 | **0.043** |  |
| HSP20-2 | 3h HS | South | 28, 32 | 3.5197 | 0.093 | 10 | **0.021** |  |
| HSP20-2 | 3h HS | North | 10, 24 | 1.9455 | 0.421 | 4 | 0.140 |  |
| HSP20-2 | 3h HS | North | 10, 28 | 8.6308 | 0.106 | 7 | **0.002** |  |
| HSP20-2 | 3h HS | North | 10, 32 | 2.1286 | 0.096 | 7 | 0.109 |  |
| HSP20-2 | 3h HS | North | 24, 28 | 8.6123 | 0.091 | 10 | **0.002** |  |
| HSP20-2 | 3h HS | North | 24, 32 | 0.93637 | 0.499 | 10 | 0.393 |  |
| HSP20-2 | 3h HS | North | 28, 32 | 8.6221 | 0.119 | 10 | **0.003** |  |
| HSP90 _443 | 3h HS | 10 | South, North | 1.03E-05 | 1 | 10 | 1 |  |
| HSP90 _443 | 3h HS | 24 | South, North | 7.1166 | 0.095 | 10 | **0.003** |  |
| HSP90 _443 | 3h HS | 28 | South, North | 1.1707 | 0.416 | 10 | 0.317 |  |
| HSP90 _443 | 3h HS | 32 | South, North | 1.9547 | 0.197 | 10 | 0.118 |  |
| HSP90 _443 | 3h HS | South | 10, 24 | 5.6372 | 0.104 | 10 | **0.003** |  |
| HSP90 _443 | 3h HS | South | 10, 28 | 8.4695 | 0.101 | 10 | **0.002** |  |
| HSP90 _443 | 3h HS | South | 10, 32 | 7.9146 | 0.117 | 10 | **0.005** |  |
| HSP90 _443 | 3h HS | South | 24, 28 | 7.3915 | 0.113 | 10 | **0.003** |  |
| HSP90 _443 | 3h HS | South | 24, 32 | 6.9564 | 0.101 | 10 | **0.005** |  |
| HSP90 _443 | 3h HS | South | 28, 32 | 0.32153 | 0.706 | 10 | 0.762 |  |
| HSP90 _443 | 3h HS | North | 10, 24 | 7.1652 | 0.102 | 10 | **0.003** |  |
| HSP90 _443 | 3h HS | North | 10, 28 | 3.1111 | 0.102 | 10 | **0.041** |  |
| HSP90 _443 | 3h HS | North | 10, 32 | 2.9508 | 0.122 | 10 | **0.033** |  |
| HSP90 _443 | 3h HS | North | 24, 28 | 1.4815 | 0.372 | 10 | 0.219 |  |
| HSP90 _443 | 3h HS | North | 24, 32 | 1.1135 | 0.403 | 10 | 0.334 |  |
| HSP90 _443 | 3h HS | North | 28, 32 | 1.8503 | 0.193 | 10 | 0.141 |  |
| HSP90 _597 | 3h HS | 10 | South, North | 1.41E-05 | 1 | 10 | 1 |  |
| HSP90 _597 | 3h HS | 24 | South, North | 0.94495 | 0.419 | 10 | 0.397 |  |
| HSP90 _597 | 3h HS | 28 | South, North | 0.80345 | 0.485 | 10 | 0.486 |  |
| HSP90 _597 | 3h HS | 32 | South, North | 7.381 | 0.118 | 10 | **0.005** |  |
| HSP90 _597 | 3h HS | South | 10, 24 | 1.2642 | 0.396 | 10 | 0.272 |  |
| HSP90 _597 | 3h HS | South | 10, 28 | 3.5234 | 0.104 | 10 | **0.033** |  |
| HSP90 _597 | 3h HS | South | 10, 32 | 7.398 | 0.097 | 10 | **0.004** |  |
| HSP90 _597 | 3h HS | South | 24, 28 | 0.98293 | 0.575 | 10 | 0.396 |  |
| HSP90 _597 | 3h HS | South | 24, 32 | 1.1087 | 0.417 | 10 | 0.333 |  |
| HSP90 _597 | 3h HS | South | 28, 32 | 5.24E-02 | 0.914 | 10 | 0.958 |  |
| HSP90 _597 | 3h HS | North | 10, 24 | 2.513 | 0.21 | 10 | 0.062 |  |
| HSP90 _597 | 3h HS | North | 10, 28 | 3.6797 | 0.104 | 10 | **0.018** |  |
| HSP90 _597 | 3h HS | North | 10, 32 | 4.1941 | 0.084 | 10 | **0.016** |  |
| HSP90 _597 | 3h HS | North | 24, 28 | 3.0051 | 0.116 | 10 | **0.040** |  |
| HSP90 _597 | 3h HS | North | 24, 32 | 1.0472 | 0.509 | 10 | 0.376 |  |
| HSP90 _597 | 3h HS | North | 28, 32 | 2.8503 | 0.116 | 10 | **0.039** |  |
| HSP90 _870 | 3h HS | 10 | South, North | 1.38E-05 | 1 | 10 | 1 |  |
| HSP90 _870 | 3h HS | 24 | South, North | 1.5366 | 0.202 | 10 | 0.203 |  |
| HSP90 _870 | 3h HS | 28 | South, North | 0.33509 | 0.803 | 10 | 0.752 |  |
| HSP90 _870 | 3h HS | 32 | South, North | 2.5751 | 0.092 | 10 | 0.061 |  |
| HSP90 _870 | 3h HS | South | 10, 24 | 1.1591 | 0.398 | 10 | 0.326 |  |
| HSP90 _870 | 3h HS | South | 10, 28 | 8.1273 | 0.085 | 10 | **0.004** |  |
| HSP90 _870 | 3h HS | South | 10, 32 | 3.4315 | 0.106 | 10 | **0.026** |  |
| HSP90 _870 | 3h HS | South | 24, 28 | 7.9037 | 0.099 | 10 | **0.002** |  |
| HSP90 _870 | 3h HS | South | 24, 32 | 1.4641 | 0.198 | 10 | 0.220 |  |
| HSP90 _870 | 3h HS | South | 28, 32 | 7.663 | 0.111 | 10 | **0.003** |  |
| HSP90 _870 | 3h HS | North | 10, 24 | 3.4476 | 0.104 | 10 | **0.025** |  |
| HSP90 _870 | 3h HS | North | 10, 28 | 3.202 | 0.108 | 10 | **0.036** |  |
| HSP90 _870 | 3h HS | North | 10, 32 | 1.3768 | 0.403 | 10 | 0.247 |  |
| HSP90 _870 | 3h HS | North | 24, 28 | 3.0295 | 0.092 | 10 | 0.051 |  |
| HSP90 _870 | 3h HS | North | 24, 32 | 3.6345 | 0.093 | 10 | **0.023** |  |
| HSP90 _870 | 3h HS | North | 28, 32 | 3.1524 | 0.093 | 10 | **0.036** |  |
| Clp_ HSP100 | Recovery | 10 | South, North | 2.42E-05 | 1 | 10 | 1 |  |
| Clp_ HSP100 | Recovery | 24 | South, North | 1.6469 | 0.208 | 10 | 0.160 |  |
| Clp_ HSP100 | Recovery | 28 | South, North | 0.7576 | 0.6 | 10 | 0.494 |  |
| Clp_ HSP100 | Recovery | 32 | South, North | 1.8349 | 0.213 | 10 | 0.144 |  |
| Clp_ HSP100 | Recovery | South | 10, 24 | 1.3884 | 0.304 | 10 | 0.230 |  |
| Clp_ HSP100 | Recovery | South | 10, 28 | 9.8971 | 0.109 | 10 | **0.002** |  |
| Clp_ HSP100 | Recovery | South | 10, 32 | 4.3027 | 0.098 | 10 | **0.012** |  |
| Clp_ HSP100 | Recovery | South | 24, 28 | 4.6606 | 0.103 | 10 | **0.010** |  |
| Clp_ HSP100 | Recovery | South | 24, 32 | 2.2501 | 0.198 | 10 | 0.092 |  |
| Clp_ HSP100 | Recovery | South | 28, 32 | 1.8299 | 0.195 | 10 | 0.144 |  |
| Clp_ HSP100 | Recovery | North | 10, 24 | 0.67384 | 0.708 | 10 | 0.553 |  |
| Clp_ HSP100 | Recovery | North | 10, 28 | 1.8876 | 0.107 | 10 | 0.139 |  |
| Clp_ HSP100 | Recovery | North | 10, 32 | 1.0841 | 0.389 | 10 | 0.351 |  |
| Clp_ HSP100 | Recovery | North | 24, 28 | 1.983 | 0.095 | 10 | 0.120 |  |
| Clp_ HSP100 | Recovery | North | 24, 32 | 1.2625 | 0.391 | 10 | 0.278 |  |
| Clp_ HSP100 | Recovery | North | 28, 32 | 1.1585 | 0.524 | 10 | 0.312 |  |
| HSP70_447 | Recovery | 10 | South, North | 9.08E-06 | 1 | 10 | 1.000 |  |
| HSP70_447 | Recovery | 24 | South, North | 1.8838 | 0.432 | 10 | 0.119 |  |
| HSP70_447 | Recovery | 28 | South, North | 13.922 | 0.093 | 10 | **0.001** |  |
| HSP70_447 | Recovery | 32 | South, North | 2.8787 | 0.090 | 10 | 0.058 |  |
| HSP70_447 | Recovery | South | 10, 24 | 2.6092 | 0.097 | 10 | 0.062 |  |
| HSP70_447 | Recovery | South | 10, 28 | 3.7119 | 0.103 | 10 | **0.021** |  |
| HSP70_447 | Recovery | South | 10, 32 | 3.1452 | 0.102 | 10 | **0.039** |  |
| HSP70_447 | Recovery | South | 24, 28 | 13.913 | 0.113 | 10 | **0.001** |  |
| HSP70_447 | Recovery | South | 24, 32 | 3.8773 | 0.116 | 10 | **0.019** |  |
| HSP70_447 | Recovery | South | 28, 32 | 2.2126 | 0.107 | 10 | 0.092 |  |
| HSP70_447 | Recovery | North | 10, 24 | 2.0234 | 0.118 | 10 | 0.114 |  |
| HSP70_447 | Recovery | North | 10, 28 | 25.134 | 0.081 | 10 | **0.001** |  |
| HSP70_447 | Recovery | North | 10, 32 | 0.14819 | 1 | 10 | 0.886 |  |
| HSP70_447 | Recovery | North | 24, 28 | 1.8923 | 0.385 | 10 | 0.128 |  |
| HSP70_447 | Recovery | North | 24, 32 | 0.82103 | 0.589 | 10 | 0.447 |  |
| HSP70_447 | Recovery | North | 28, 32 | 1.538 | 0.102 | 10 | 0.193 |  |
| HSP70_696 | Recovery | 10 | South, North | 1.26E-04 | 1 | 10 | 1 |  |
| HSP70_696 | Recovery | 24 | South, North | 2.0143 | 0.087 | 10 | 0.112 |  |
| HSP70_696 | Recovery | 28 | South, North | 1.0434 | 0.595 | 10 | 0.354 |  |
| HSP70_696 | Recovery | 32 | South, North | 0.71312 | 0.799 | 10 | 0.533 |  |
| HSP70_696 | Recovery | South | 10, 24 | 13.056 | 0.096 | 10 | **0.003** |  |
| HSP70_696 | Recovery | South | 10, 28 | 11.753 | 0.087 | 10 | **0.001** |  |
| HSP70_696 | Recovery | South | 10, 32 | 7.7574 | 0.108 | 10 | **0.004** |  |
| HSP70_696 | Recovery | South | 24, 28 | 0.46501 | 1 | 10 | 0.675 |  |
| HSP70_696 | Recovery | South | 24, 32 | 3.6799 | 0.102 | 10 | **0.028** |  |
| HSP70_696 | Recovery | South | 28, 32 | 3.117 | 0.099 | 10 | **0.039** |  |
| HSP70_696 | Recovery | North | 10, 24 | 29.979 | 0.120 | 10 | **0.001** |  |
| HSP70_696 | Recovery | North | 10, 28 | 10.339 | 0.085 | 10 | **0.001** |  |
| HSP70_696 | Recovery | North | 10, 32 | 1.3283 | 0.385 | 10 | 0.252 |  |
| HSP70_696 | Recovery | North | 24, 28 | 4.1079 | 0.101 | 10 | **0.018** |  |
| HSP70_696 | Recovery | North | 24, 32 | 2.5904 | 0.109 | 10 | **0.061** |  |
| HSP70_696 | Recovery | North | 28, 32 | 1.4661 | 0.167 | 10 | 0.222 |  |
| HSP20-2 | Recovery | 10 | South, North | 1.03E-05 | 1 | 10 | 1 |  |
| HSP20-2 | Recovery | 24 | South, North | 20.559 | 0.095 | 10 | **0.001** |  |
| HSP20-2 | Recovery | 28 | South, North | 1.1329 | 0.398 | 10 | 0.311 |  |
| HSP20-2 | Recovery | 32 | South, North | 1.3248 | 0.193 | 10 | 0.268 |  |
| HSP20-2 | Recovery | South | 10, 24 | 7.2648 | 0.098 | 10 | **0.002** |  |
| HSP20-2 | Recovery | South | 10, 28 | 7.3946 | 0.107 | 10 | **0.002** |  |
| HSP20-2 | Recovery | South | 10, 32 | 5.7349 | 0.102 | 10 | **0.002** |  |
| HSP20-2 | Recovery | South | 24, 28 | 1.7985 | 0.194 | 10 | 0.149 |  |
| HSP20-2 | Recovery | South | 24, 32 | 0.52922 | 0.613 | 10 | 0.602 |  |
| HSP20-2 | Recovery | South | 28, 32 | 0.47399 | 0.587 | 10 | 0.665 |  |
| HSP20-2 | Recovery | North | 10, 24 | 3.1647 | 0.124 | 10 | **0.030** |  |
| HSP20-2 | Recovery | North | 10, 28 | 2.5533 | 0.097 | 10 | 0.061 |  |
| HSP20-2 | Recovery | North | 10, 32 | 0.10549 | 0.805 | 10 | 0.912 |  |
| HSP20-2 | Recovery | North | 24, 28 | 5.9466 | 0.117 | 10 | **0.006** |  |
| HSP20-2 | Recovery | North | 24, 32 | 1.8611 | 0.084 | 10 | 0.141 |  |
| HSP20-2 | Recovery | North | 28, 32 | 1.5293 | 0.101 | 10 | 0.222 |  |
| HSP90 _443 | Recovery | 10 | South, North | Negative |  |  |  |  |
| HSP90 _443 | Recovery | 24 | South, North | 1.3298 | 0.425 | 10 | 0.233 |  |
| HSP90 _443 | Recovery | 28 | South, North | 2.8143 | 0.106 | 10 | **0.049** |  |
| HSP90 _443 | Recovery | 32 | South, North | 1.5911 | 0.387 | 10 | 0.197 |  |
| HSP90 _443 | Recovery | South | 10, 24 | 2.205 | 0.204 | 10 | 0.094 |  |
| HSP90 _443 | Recovery | South | 10, 28 | 3.5265 | 0.109 | 10 | **0.024** |  |
| HSP90 _443 | Recovery | South | 10, 32 | 0.60926 | 0.585 | 10 | 0.587 |  |
| HSP90 _443 | Recovery | South | 24, 28 | 5.6818 | 0.104 | 10 | **0.007** |  |
| HSP90 _443 | Recovery | South | 24, 32 | 0.29967 | 1 | 10 | 0.822 |  |
| HSP90 _443 | Recovery | South | 28, 32 | 1.7476 | 0.280 | 10 | 0.146 |  |
| HSP90 _443 | Recovery | North | 10, 24 | 0.25853 | 0.705 | 10 | 0.800 |  |
| HSP90 _443 | Recovery | North | 10, 28 | 0.46794 | 0.685 | 10 | 0.670 |  |
| HSP90 _443 | Recovery | North | 10, 32 | 2.1996 | 0.122 | 10 | 0.084 |  |
| HSP90 _443 | Recovery | North | 24, 28 | 7.30E-03 | 1 | 10 | 0.995 |  |
| HSP90 _443 | Recovery | North | 24, 32 | 0.87236 | 0.524 | 10 | 0.431 |  |
| HSP90 _443 | Recovery | North | 28, 32 | 2.3527 | 0.186 | 10 | 0.081 |  |
| HSP90 _597 | Recovery | 10 | South, North | Negative |  |  |  |  |
| HSP90 _597 | Recovery | 24 | South, North | 0.29598 | 1 | 10 | 0.789 |  |
| HSP90 _597 | Recovery | 28 | South, North | 6.0364 | 0.097 | 10 | **0.006** |  |
| HSP90 _597 | Recovery | 32 | South, North | 5.5027 | 0.095 | 10 | **0.007** |  |
| HSP90 _597 | Recovery | South | 10, 24 | 1.1716 | 0.383 | 10 | 0.307 |  |
| HSP90 _597 | Recovery | South | 10, 28 | 4.5953 | 0.098 | 10 | **0.012** |  |
| HSP90 _597 | Recovery | South | 10, 32 | 1.7262 | 0.306 | 10 | 0.183 |  |
| HSP90 _597 | Recovery | South | 24, 28 | 9.53E-03 | 1 | 10 | 0.990 |  |
| HSP90 _597 | Recovery | South | 24, 32 | 1.9552 | 0.204 | 10 | 0.120 |  |
| HSP90 _597 | Recovery | South | 28, 32 | 3.7777 | 0.090 | 10 | **0.026** |  |
| HSP90 _597 | Recovery | North | 10, 24 | 3.488 | 0.088 | 10 | **0.026** |  |
| HSP90 _597 | Recovery | North | 10, 28 | 12.4 | 0.101 | 10 | **0.001** |  |
| HSP90 _597 | Recovery | North | 10, 32 | 7.3342 | 0.115 | 10 | **0.003** |  |
| HSP90 _597 | Recovery | North | 24, 28 | 2.1136 | 0.090 | 10 | 0.095 |  |
| HSP90 _597 | Recovery | North | 24, 32 | 2.0358 | 0.197 | 10 | 0.107 |  |
| HSP90 _597 | Recovery | North | 28, 32 | 0.44434 | 0.819 | 10 | 0.680 |  |
| HSP90 _870 | Recovery | 10 | South, North | Negative |  |  |  |  |
| HSP90 _870 | Recovery | 24 | South, North | 0.17516 | 1 | 10 | 0.854 |  |
| HSP90 _870 | Recovery | 28 | South, North | 0.67991 | 0.489 | 10 | 0.563 |  |
| HSP90 _870 | Recovery | 32 | South, North | 10.543 | 0.104 | 10 | **0.001** |  |
| HSP90 _870 | Recovery | South | 10, 24 | 10.287 | 0.088 | 10 | **0.001** |  |
| HSP90 _870 | Recovery | South | 10, 28 | 8.7192 | 0.110 | 10 | **0.002** |  |
| HSP90 _870 | Recovery | South | 10, 32 | 9.3324 | 0.092 | 10 | **0.001** |  |
| HSP90 _870 | Recovery | South | 24, 28 | 3.8873 | 0.101 | 10 | **0.018** |  |
| HSP90 _870 | Recovery | South | 24, 32 | 4.2321 | 0.091 | 10 | **0.011** |  |
| HSP90 _870 | Recovery | South | 28, 32 | 0.54144 | 0.611 | 10 | 0.618 |  |
| HSP90 _870 | Recovery | North | 10, 24 | 6.9611 | 0.092 | 10 | **0.006** |  |
| HSP90 _870 | Recovery | North | 10, 28 | 5.5532 | 0.083 | 10 | **0.004** |  |
| HSP90 _870 | Recovery | North | 10, 32 | 1.2305 | 0.310 | 10 | 0.264 |  |
| HSP90 _870 | Recovery | North | 24, 28 | 4.8281 | 0.093 | 10 | **0.008** |  |
| HSP90 _870 | Recovery | North | 24, 32 | 11.422 | 0.109 | 10 | **0.001** |  |
| HSP90 _870 | Recovery | North | 28, 32 | 9.1957 | 0.104 | 10 | **0.002** |  |

**Supplementary Table F** – *Fv/Fm* raw data

| **Time** | **Species** | **Edge** | **Temp** | **replicate** | ***Fv/Fm*** | ***Fv/Fm*** | **mean *Fv/Fm*** |
| --- | --- | --- | --- | --- | --- | --- | --- |
| 0 | Fv | pt | 10 | 1 | 0.762 | 0.781 | 0.772 |
| 0 | Fv | pt | 10 | 2 | 0.776 | 0.758 | 0.767 |
| 0 | Fv | pt | 10 | 3 | 0.756 | 0.718 | 0.737 |
| 0 | Fv | pt | 10 | 4 | 0.750 | 0.761 | 0.756 |
| 0 | Fv | pt | 10 | 5 | 0.766 | 0.772 | 0.769 |
| 0 | Fv | gr | 10 | 1 | 0.703 | 0.703 | 0.703 |
| 0 | Fv | gr | 10 | 2 | 0.715 | 0.726 | 0.721 |
| 0 | Fv | gr | 10 | 3 | 0.646 | 0.717 | 0.682 |
| 0 | Fv | gr | 10 | 4 | 0.713 | 0.719 | 0.716 |
| 0 | Fv | gr | 10 | 5 | 0.732 | 0.713 | 0.723 |
| 0 | Zm | pt | 10 | 1 | 0.797 | 0.790 | 0.794 |
| 0 | Zm | pt | 10 | 2 | 0.792 | 0.813 | 0.803 |
| 0 | Zm | pt | 10 | 3 | 0.715 | 0.697 | 0.706 |
| 0 | Zm | pt | 10 | 4 | 0.763 | 0.763 | 0.763 |
| 0 | Zm | pt | 10 | 5 | 0.788 | 0.768 | 0.778 |
| 0 | Zm | gr | 10 | 1 | 0.744 | 0.766 | 0.755 |
| 0 | Zm | gr | 10 | 2 | 0.771 | 0.797 | 0.784 |
| 0 | Zm | gr | 10 | 3 | 0.744 | 0.737 | 0.741 |
| 0 | Zm | gr | 10 | 4 | 0.766 | 0.780 | 0.773 |
| 0 | Zm | gr | 10 | 5 | 0.782 | 0.780 | 0.781 |
| 3 | Fv | pt | 18 | 1 | 0.735 | 0.720 | 0.728 |
| 3 | Fv | pt | 18 | 2 | 0.797 | 0.701 | 0.749 |
| 3 | Fv | pt | 18 | 3 | 0.778 | 0.736 | 0.757 |
| 3 | Fv | pt | 18 | 4 | 0.787 | 0.797 | 0.792 |
| 3 | Fv | pt | 18 | 5 | 0.771 | 0.749 | 0.760 |
| 3 | Fv | gr | 18 | 1 | 0.688 | 0.694 | 0.691 |
| 3 | Fv | gr | 18 | 2 | 0.687 | 0.711 | 0.699 |
| 3 | Fv | gr | 18 | 3 | 0.657 | 0.696 | 0.677 |
| 3 | Fv | gr | 18 | 4 | 0.647 | 0.656 | 0.652 |
| 3 | Fv | gr | 18 | 5 | 0.720 | 0.684 | 0.702 |
| 3 | Fv | pt | 24 | 1 | 0.743 | 0.757 | 0.750 |
| 3 | Fv | pt | 24 | 2 | 0.746 | 0.747 | 0.747 |
| 3 | Fv | pt | 24 | 3 | 0.761 | 0.733 | 0.747 |
| 3 | Fv | pt | 24 | 4 | 0.729 | 0.751 | 0.740 |
| 3 | Fv | pt | 24 | 5 | 0.707 | 0.756 | 0.732 |
| 3 | Fv | gr | 24 | 1 | 0.631 | 0.654 | 0.643 |
| 3 | Fv | gr | 24 | 2 | 0.681 | 0.642 | 0.662 |
| 3 | Fv | gr | 24 | 3 | 0.676 | 0.693 | 0.685 |
| 3 | Fv | gr | 24 | 4 | 0.751 | 0.698 | 0.725 |
| 3 | Fv | gr | 24 | 5 | 0.623 | 0.691 | 0.657 |
| 3 | Fv | pt | 28 | 1 | 0.649 | 0.725 | 0.687 |
| 3 | Fv | pt | 28 | 2 | 0.706 | 0.736 | 0.721 |
| 3 | Fv | pt | 28 | 3 | 0.788 | 0.794 | 0.791 |
| 3 | Fv | pt | 28 | 4 | 0.727 | 0.756 | 0.742 |
| 3 | Fv | pt | 28 | 5 | 0.775 | 0.786 | 0.781 |
| 3 | Fv | gr | 28 | 1 | 0.610 | 0.577 | 0.594 |
| 3 | Fv | gr | 28 | 2 | 0.638 | 0.576 | 0.607 |
| 3 | Fv | gr | 28 | 3 | 0.709 | 0.665 | 0.687 |
| 3 | Fv | gr | 28 | 4 | 0.625 | 0.625 | 0.625 |
| 3 | Fv | gr | 28 | 5 | 0.643 | 0.606 | 0.625 |
| 3 | Fv | pt | 32 | 1 | 0.690 | 0.670 | 0.680 |
| 3 | Fv | pt | 32 | 2 | 0.659 | 0.528 | 0.594 |
| 3 | Fv | pt | 32 | 3 | 0.675 | 0.641 | 0.658 |
| 3 | Fv | pt | 32 | 4 | 0.482 | 0.716 | 0.599 |
| 3 | Fv | pt | 32 | 5 | 0.764 | 0.639 | 0.702 |
| 3 | Fv | gr | 32 | 1 | 0.331 | 0.377 | 0.354 |
| 3 | Fv | gr | 32 | 2 | 0.500 | 0.330 | 0.415 |
| 3 | Fv | gr | 32 | 3 | 0.425 | 0.599 | 0.512 |
| 3 | Fv | gr | 32 | 4 | 0.398 | 0.393 | 0.396 |
| 3 | Fv | gr | 32 | 5 | 0.510 | 0.429 | 0.470 |
| 24 | Fv | pt | 18 | 1 | 0.733 | 0.768 | 0.751 |
| 24 | Fv | pt | 18 | 2 | 0.766 | 0.732 | 0.749 |
| 24 | Fv | pt | 18 | 3 | 0.755 | 0.661 | 0.708 |
| 24 | Fv | pt | 18 | 4 | 0.750 | 0.779 | 0.765 |
| 24 | Fv | pt | 18 | 5 | 0.744 | 0.746 | 0.745 |
| 24 | Fv | gr | 18 | 1 | 0.681 | 0.725 | 0.703 |
| 24 | Fv | gr | 18 | 2 | 0.717 | 0.674 | 0.696 |
| 24 | Fv | gr | 18 | 3 | 0.708 | 0.717 | 0.713 |
| 24 | Fv | gr | 18 | 4 | 0.730 | 0.758 | 0.744 |
| 24 | Fv | gr | 18 | 5 | 0.726 | 0.700 | 0.713 |
| 24 | Fv | pt | 24 | 1 | 0.734 | 0.753 | 0.744 |
| 24 | Fv | pt | 24 | 2 | 0.767 | 0.767 | 0.767 |
| 24 | Fv | pt | 24 | 3 | 0.784 | 0.772 | 0.778 |
| 24 | Fv | pt | 24 | 4 | 0.776 | 0.758 | 0.767 |
| 24 | Fv | pt | 24 | 5 | 0.771 | 0.786 | 0.779 |
| 24 | Fv | gr | 24 | 1 | 0.701 | 0.682 | 0.692 |
| 24 | Fv | gr | 24 | 2 | 0.708 | 0.705 | 0.707 |
| 24 | Fv | gr | 24 | 3 | 0.729 | 0.734 | 0.732 |
| 24 | Fv | gr | 24 | 4 | 0.734 | 0.707 | 0.721 |
| 24 | Fv | gr | 24 | 5 | 0.734 | 0.713 | 0.724 |
| 24 | Fv | pt | 28 | 1 | 0.766 | 0.792 | 0.779 |
| 24 | Fv | pt | 28 | 2 | 0.774 | 0.803 | 0.789 |
| 24 | Fv | pt | 28 | 3 | 0.761 | 0.786 | 0.774 |
| 24 | Fv | pt | 28 | 4 | 0.778 | 0.720 | 0.749 |
| 24 | Fv | pt | 28 | 5 | 0.763 | 0.718 | 0.741 |
| 24 | Fv | gr | 28 | 1 | 0.674 | 0.756 | 0.715 |
| 24 | Fv | gr | 28 | 2 | 0.708 | 0.729 | 0.719 |
| 24 | Fv | gr | 28 | 3 | 0.709 | 0.752 | 0.731 |
| 24 | Fv | gr | 28 | 4 | 0.723 | 0.721 | 0.722 |
| 24 | Fv | gr | 28 | 5 | 0.694 | 0.708 | 0.701 |
| 24 | Fv | pt | 32 | 1 | 0.708 | 0.767 | 0.738 |
| 24 | Fv | pt | 32 | 2 | 0.732 | 0.749 | 0.741 |
| 24 | Fv | pt | 32 | 3 | 0.756 | 0.747 | 0.752 |
| 24 | Fv | pt | 32 | 4 | 0.763 | 0.734 | 0.749 |
| 24 | Fv | pt | 32 | 5 | 0.760 | 0.685 | 0.723 |
| 24 | Fv | gr | 32 | 1 | 0.476 | 0.481 | 0.479 |
| 24 | Fv | gr | 32 | 2 | 0.439 | 0.450 | 0.445 |
| 24 | Fv | gr | 32 | 3 | 0.581 | 0.625 | 0.603 |
| 24 | Fv | gr | 32 | 4 | 0.539 | 0.658 | 0.599 |
| 24 | Fv | gr | 32 | 5 | 0.494 | 0.670 | 0.582 |
| 3 | Fv | pt | 10 | 1 | 0.727 | 0.748 | 0.738 |
| 3 | Fv | pt | 10 | 2 | 0.747 | 0.759 | 0.753 |
| 3 | Fv | pt | 10 | 3 | 0.729 | 0.758 | 0.744 |
| 3 | Fv | pt | 10 | 4 | 0.764 | 0.751 | 0.758 |
| 3 | Fv | pt | 10 | 5 | 0.759 | 0.749 | 0.754 |
| 3 | Fv | gr | 10 | 1 | 0.697 | 0.700 | 0.699 |
| 3 | Fv | gr | 10 | 2 | 0.703 | 0.689 | 0.696 |
| 3 | Fv | gr | 10 | 3 | 0.741 | 0.710 | 0.726 |
| 3 | Fv | gr | 10 | 4 | 0.720 | 0.720 | 0.720 |
| 3 | Fv | gr | 10 | 5 | 0.733 | 0.709 | 0.721 |
| 24 | Fv | pt | 10 | 1 | 0.748 | 0.775 | 0.762 |
| 24 | Fv | pt | 10 | 2 | 0.736 | 0.789 | 0.763 |
| 24 | Fv | pt | 10 | 3 | 0.795 | 0.777 | 0.786 |
| 24 | Fv | pt | 10 | 4 | 0.778 | 0.773 | 0.776 |
| 24 | Fv | pt | 10 | 5 | 0.766 | 0.775 | 0.771 |
| 24 | Fv | gr | 10 | 1 | 0.699 | 0.717 | 0.708 |
| 24 | Fv | gr | 10 | 2 | 0.732 | 0.732 | 0.732 |
| 24 | Fv | gr | 10 | 3 | 0.721 | 0.747 | 0.734 |
| 24 | Fv | gr | 10 | 4 | 0.744 | 0.729 | 0.737 |
| 24 | Fv | gr | 10 | 5 | 0.755 | 0.776 | 0.766 |
| 3 | Zm | pt | 18 | 1 | 0.703 | 0.798 | 0.751 |
| 3 | Zm | pt | 18 | 2 | 0.817 | 0.765 | 0.791 |
| 3 | Zm | pt | 18 | 3 | 0.663 | 0.706 | 0.685 |
| 3 | Zm | pt | 18 | 4 | 0.802 | 0.759 | 0.781 |
| 3 | Zm | pt | 18 | 5 | 0.810 | 0.800 | 0.805 |
| 3 | Zm | gr | 18 | 1 | 0.785 | 0.780 | 0.783 |
| 3 | Zm | gr | 18 | 2 | 0.780 | 0.760 | 0.770 |
| 3 | Zm | gr | 18 | 3 | 0.754 | 0.764 | 0.759 |
| 3 | Zm | gr | 18 | 4 | 0.776 | 0.795 | 0.786 |
| 3 | Zm | gr | 18 | 5 | 0.793 | 0.733 | 0.763 |
| 3 | Zm | pt | 24 | 1 | 0.742 | 0.727 | 0.735 |
| 3 | Zm | pt | 24 | 2 | 0.722 | 0.767 | 0.745 |
| 3 | Zm | pt | 24 | 3 | 0.788 | 0.784 | 0.786 |
| 3 | Zm | pt | 24 | 4 | 0.742 | 0.724 | 0.733 |
| 3 | Zm | pt | 24 | 5 | 0.400 | 0.738 | 0.569 |
| 3 | Zm | gr | 24 | 1 | 0.718 | 0.698 | 0.708 |
| 3 | Zm | gr | 24 | 2 | 0.747 | 0.787 | 0.767 |
| 3 | Zm | gr | 24 | 3 | 0.761 | 0.757 | 0.759 |
| 3 | Zm | gr | 24 | 4 | 0.688 | 0.748 | 0.718 |
| 3 | Zm | gr | 24 | 5 | 0.716 | 0.667 | 0.692 |
| 3 | Zm | pt | 28 | 1 | 0.730 | 0.761 | 0.746 |
| 3 | Zm | pt | 28 | 2 | 0.795 | 0.781 | 0.788 |
| 3 | Zm | pt | 28 | 3 | 0.811 | 0.771 | 0.791 |
| 3 | Zm | pt | 28 | 4 | 0.739 | 0.699 | 0.719 |
| 3 | Zm | pt | 28 | 5 | 0.795 | 0.798 | 0.797 |
| 3 | Zm | gr | 28 | 1 | 0.700 | 0.740 | 0.720 |
| 3 | Zm | gr | 28 | 2 | 0.784 | 0.756 | 0.770 |
| 3 | Zm | gr | 28 | 3 | 0.710 | 0.810 | 0.760 |
| 3 | Zm | gr | 28 | 4 | 0.776 | 0.788 | 0.782 |
| 3 | Zm | gr | 28 | 5 | 0.770 | 0.722 | 0.746 |
| 3 | Zm | pt | 32 | 1 | 0.692 | 0.625 | 0.659 |
| 3 | Zm | pt | 32 | 2 | 0.734 | 0.739 | 0.737 |
| 3 | Zm | pt | 32 | 3 | 0.775 | 0.735 | 0.755 |
| 3 | Zm | pt | 32 | 4 | 0.772 | 0.768 | 0.770 |
| 3 | Zm | pt | 32 | 5 | 0.420 | 0.625 | 0.523 |
| 3 | Zm | gr | 32 | 1 | 0.662 | 0.751 | 0.707 |
| 3 | Zm | gr | 32 | 2 | 0.757 | 0.567 | 0.662 |
| 3 | Zm | gr | 32 | 3 | 0.718 | 0.716 | 0.717 |
| 3 | Zm | gr | 32 | 4 | 0.657 | 0.722 | 0.690 |
| 3 | Zm | gr | 32 | 5 | 0.725 | 0.758 | 0.742 |
| 24 | Zm | pt | 18 | 1 | 0.793 | 0.781 | 0.787 |
| 24 | Zm | pt | 18 | 2 | 0.757 | 0.752 | 0.755 |
| 24 | Zm | pt | 18 | 3 | 0.759 | 0.769 | 0.764 |
| 24 | Zm | pt | 18 | 4 | 0.789 | 0.799 | 0.794 |
| 24 | Zm | pt | 18 | 5 | 0.761 | 0.790 | 0.776 |
| 24 | Zm | gr | 18 | 1 | 0.765 | 0.811 | 0.788 |
| 24 | Zm | gr | 18 | 2 | 0.806 | 0.782 | 0.794 |
| 24 | Zm | gr | 18 | 3 | 0.759 | 0.777 | 0.768 |
| 24 | Zm | gr | 18 | 4 | 0.705 | 0.790 | 0.748 |
| 24 | Zm | gr | 18 | 5 | 0.753 | 0.747 | 0.750 |
| 24 | Zm | pt | 24 | 1 | 0.788 | 0.835 | 0.812 |
| 24 | Zm | pt | 24 | 2 | 0.798 | 0.789 | 0.794 |
| 24 | Zm | pt | 24 | 3 | 0.808 | 0.814 | 0.811 |
| 24 | Zm | pt | 24 | 4 | 0.695 | 0.730 | 0.713 |
| 24 | Zm | pt | 24 | 5 | 0.551 | 0.474 | 0.513 |
| 24 | Zm | gr | 24 | 1 | 0.761 | 0.797 | 0.779 |
| 24 | Zm | gr | 24 | 2 | 0.768 | 0.784 | 0.776 |
| 24 | Zm | gr | 24 | 3 | 0.711 | 0.746 | 0.729 |
| 24 | Zm | gr | 24 | 4 | 0.774 | 0.744 | 0.759 |
| 24 | Zm | gr | 24 | 5 | 0.747 | 0.791 | 0.769 |
| 24 | Zm | pt | 28 | 1 | 0.816 | 0.786 | 0.801 |
| 24 | Zm | pt | 28 | 2 | 0.653 | nd | 0.653 |
| 24 | Zm | pt | 28 | 3 | 0.560 | 0.622 | 0.591 |
| 24 | Zm | pt | 28 | 4 | 0.812 | 0.825 | 0.819 |
| 24 | Zm | pt | 28 | 5 | 0.814 | 0.768 | 0.791 |
| 24 | Zm | gr | 28 | 1 | 0.660 | 0.792 | 0.726 |
| 24 | Zm | gr | 28 | 2 | 0.761 | 0.746 | 0.754 |
| 24 | Zm | gr | 28 | 3 | 0.783 | 0.741 | 0.762 |
| 24 | Zm | gr | 28 | 4 | 0.784 | 0.802 | 0.793 |
| 24 | Zm | gr | 28 | 5 | 0.780 | 0.776 | 0.778 |
| 24 | Zm | pt | 32 | 1 | 0.642 | 0.667 | 0.655 |
| 24 | Zm | pt | 32 | 2 | 0.785 | 0.763 | 0.774 |
| 24 | Zm | pt | 32 | 3 | 0.757 | 0.778 | 0.768 |
| 24 | Zm | pt | 32 | 4 | 0.764 | 0.757 | 0.761 |
| 24 | Zm | pt | 32 | 5 | 0.791 | 0.691 | 0.741 |
| 24 | Zm | gr | 32 | 1 | 0.700 | 0.733 | 0.717 |
| 24 | Zm | gr | 32 | 2 | 0.739 | 0.756 | 0.748 |
| 24 | Zm | gr | 32 | 3 | 0.547 | 0.626 | 0.587 |
| 24 | Zm | gr | 32 | 4 | 0.715 | 0.721 | 0.718 |
| 24 | Zm | gr | 32 | 5 | 0.653 | 0.733 | 0.693 |
| 3 | Zm | pt | 10 | 1 | 0.699 | 0.731 | 0.715 |
| 3 | Zm | pt | 10 | 2 | 0.643 | 0.770 | 0.707 |
| 3 | Zm | pt | 10 | 3 | 0.732 | 0.737 | 0.735 |
| 3 | Zm | pt | 10 | 4 | 0.774 | 0.773 | 0.774 |
| 3 | Zm | pt | 10 | 5 | 0.815 | 0.829 | 0.822 |
| 3 | Zm | gr | 10 | 1 | 0.752 | 0.775 | 0.764 |
| 3 | Zm | gr | 10 | 2 | 0.748 | 0.763 | 0.756 |
| 3 | Zm | gr | 10 | 3 | 0.793 | 0.726 | 0.760 |
| 3 | Zm | gr | 10 | 4 | 0.768 | 0.787 | 0.778 |
| 3 | Zm | gr | 10 | 5 | 0.775 | 0.670 | 0.723 |
| 24 | Zm | pt | 10 | 1 | 0.810 | 0.854 | 0.832 |
| 24 | Zm | pt | 10 | 2 | 0.809 | 0.849 | 0.829 |
| 24 | Zm | pt | 10 | 3 | 0.844 | 0.840 | 0.842 |
| 24 | Zm | pt | 10 | 4 | 0.820 | 0.761 | 0.791 |
| 24 | Zm | pt | 10 | 5 | nd | 0.805 | 0.805 |
| 24 | Zm | gr | 10 | 1 | 0.835 | 0.754 | 0.795 |
| 24 | Zm | gr | 10 | 2 | 0.760 | 0.776 | 0.768 |
| 24 | Zm | gr | 10 | 3 | 0.826 | 0.795 | 0.811 |
| 24 | Zm | gr | 10 | 4 | 0.775 | 0.620 | 0.698 |
| 24 | Zm | gr | 10 | 5 | 0.800 | 0.793 | 0.797 |

**Supplementary Table G** – Relative gene expression data of the technical duplicates normalised to the geometric average of the three reference genes. Each sample was amplified in duplicate qPCR reactions, on an iCycler iQ Detection System (BioRad), with a total volume of 20 μL using a SYBR green-based detection kit (PerfeCta Fastmix for iQ, Quanta biosciences), 5 μL of cDNA template from a 10^–2^ dilution and 0.25 μM of each primer. Cycle parameters were of 95 °C for 3 min and then 40 cycles at 95 °C for 10 s and 68 °C for 30 s. The resulting OPD files were analysed using iQ5 software (BioRad), with manual threshold settings and efficiency correction. For each gene, the PCR Efficiency was determined from a calibration curve, using a dilution series of pooled cDNA from all samples. The resulting individual expression values, normalised to the geometric average of the (EF1, Sumo3, and tubulin) were then normalised to the mean value of each reference condition (10ºC) for further analysis.

| **Edge** | **Temp** | **Time** | **sample** | **Clp_ HSP100** | **HSP70_447** | **HSP90 _597** | **HSP70_696** | **HSP90 _870** | **HSP20-2** | **HSP90 _443** |
| --- | --- | --- | --- | --- | --- | --- | --- | --- | --- | --- |
| North | 10 | 3h HS | Q25 | 3.295 | 2.429 | 1.515 | 1.443 | 1.572 | 1.09 | 1.891 |
| North | 10 | 3h HS | Q27 | 0.459 | 0.705 | 0.929 | 0.844 | 0.392 | 2.73 | 1.010 |
| North | 10 | 3h HS | Q26 | 0.480 | 0.517 | 0.630 | 0.799 | 0.925 | 0.55 | 0.066 |
| North | 10 | 3h HS | Q25 | 0.972 | 0.549 | 1.124 | 1.269 | 1.861 | 0.55 | 2.230 |
| North | 10 | 3h HS | Q26 | 0.423 | 0.755 | 0.830 | 0.693 | 0.935 | 0.55 | 0.072 |
| North | 10 | 3h HS | Q27 | 0.371 | 1.044 | 0.972 | 0.951 | 0.314 | 0.55 | 0.732 |
| North | 10 | Recovery | Q23 | 0.789 | 2.733 | 2.117 | 0.387 | 2.365 | 162.55 | 7.609 |
| North | 10 | Recovery | Q24 | 1.211 | 0.906 | 0.780 | 0.373 | 2.164 | 0.00 | 3.329 |
| North | 10 | Recovery | Q22 | 1.047 | 4.024 | 0.910 | 0.407 | 1.653 | 0.00 | 3.667 |
| North | 10 | Recovery | Q22 | 1.440 | 3.914 | 1.447 | 0.399 | 1.613 | 1.09 | 4.667 |
| North | 10 | Recovery | Q24 | 1.410 | 0.994 | 0.921 | 0.441 | 2.219 | 0.55 | 3.204 |
| North | 10 | Recovery | Q23 | 1.121 | 2.054 | 2.070 | 0.451 | 2.123 | 129.82 | 8.259 |
| North | 24 | 3h HS | Q28 | 20.454 | 49.895 | 5.437 | 5.185 | 9.410 | 50841.82 | 3.707 |
| North | 24 | 3h HS | Q30 | 3.135 | 10.151 | 2.678 | 3.101 | 7.766 | 4986.00 | 4.323 |
| North | 24 | 3h HS | Q29 | 46.686 | 271.786 | 1.161 | 11.260 | 35.463 | 39513.82 | 15.273 |
| North | 24 | 3h HS | Q28 | 4.568 | 13.717 | 4.006 | 3.774 | 9.986 | 3429.82 | 5.509 |
| North | 24 | 3h HS | Q29 | 49.931 | 273.386 | 1.866 | 14.254 | 48.780 | 33356.18 | 20.108 |
| North | 24 | 3h HS | Q30 | 3.000 | 12.690 | 2.858 | 3.307 | 8.154 | 4759.09 | 4.757 |
| North | 24 | Recovery | Q38 | 1.502 | 2.746 | 1.402 | 0.540 | 1.016 | 78.00 | 1.007 |
| North | 24 | Recovery | Q39 | 0.873 | 0.393 | 1.454 | 0.648 | 1.633 | 3.82 | 5.013 |
| North | 24 | Recovery | Q37 | 1.221 | 1.634 | 1.448 | 0.529 | 1.362 | 6.55 | 5.687 |
| North | 24 | Recovery | Q37 | 0.745 | 1.372 | 1.600 | 0.558 | 1.264 | 1.64 | 5.976 |
| North | 24 | Recovery | Q39 | 1.078 | 0.371 | 1.595 | 0.546 | 1.594 | 9.82 | 4.595 |
| North | 24 | Recovery | Q38 | 1.018 | 2.261 | 1.392 | 0.570 | 0.969 | 43.09 | 1.358 |
| North | 28 | 3h HS | Q41 | 2.977 | 73.394 | 2.547 | 3.597 | 4.699 | 168.55 | 5.811 |
| North | 28 | 3h HS | Q43 | 2.777 | 67.370 | 2.302 | 4.234 | 19.980 | 33943.09 | 0.364 |
| North | 28 | 3h HS | Q42 | 2.531 | 53.360 | 1.722 | 4.694 | 12.499 | 14837.45 | 5.379 |
| North | 28 | 3h HS | Q41 | 2.370 | 83.800 | 2.692 | 3.709 | 10.100 | 183.27 | 7.217 |
| North | 28 | 3h HS | Q42 | 4.699 | 56.090 | 2.388 | 3.825 | 13.309 | 13072.36 | 4.922 |
| North | 28 | 3h HS | Q43 | 2.455 | 68.034 | 1.815 | 4.064 | 19.611 | 38228.18 | 0.427 |
| North | 28 | Recovery | Q45 | 5.136 | 95.691 | 1.353 | 0.945 | 2.021 | 492.00 | 1.653 |
| North | 28 | Recovery | Q46 | 2.334 | 0.612 | 0.663 | 0.700 | 1.822 | 119.45 | 0.567 |
| North | 28 | Recovery | Q44 | 1.286 | 1.262 | 1.958 | 0.573 | 1.164 | 6.00 | 8.114 |
| North | 28 | Recovery | Q44 | 1.900 | 1.458 | 1.653 | 0.638 | 1.335 | 9.82 | 7.735 |
| North | 28 | Recovery | Q46 | 3.101 | 0.699 | 0.761 | 0.554 | 2.195 | 110.73 | 0.469 |
| North | 28 | Recovery | Q45 | 5.101 | 102.856 | 1.414 | 0.870 | 3.342 | 202.91 | 1.619 |
| North | 32 | 3h HS | Q48 | 0.583 | 3.698 | 0.873 | 1.973 | 1.757 | 5362.91 | 2.993 |
| North | 32 | 3h HS | Q50 | 0.734 | 3.367 | 0.859 | 2.122 | 6.110 | 3274.91 | 5.807 |
| North | 32 | 3h HS | Q49 | 0.219 | 1.499 | 0.350 | 0.795 | 3.971 | 838.91 | 0.184 |
| North | 32 | 3h HS | Q48 | 0.942 | 4.674 | 0.861 | 1.928 | 5.538 | 5074.91 | 3.608 |
| North | 32 | 3h HS | Q49 | 0.293 | 1.899 | 0.621 | 0.789 | 3.765 | 1129.09 | 0.272 |
| North | 32 | 3h HS | Q50 | 0.882 | 3.080 | 0.884 | 2.146 | 5.765 | 4096.91 | 7.398 |
| North | 32 | Recovery | Q34 | 1.912 | 34.050 | 0.373 | 1.997 | 11.452 | 9276.00 | 0.968 |
| North | 32 | Recovery | Q35 | 0.802 | 20.357 | 0.503 | 2.015 | 9.769 | 8646.00 | 0.743 |
| North | 32 | Recovery | Q40 | 2.848 | 176.197 | 0.999 | 4.492 | 12.677 | 39164.18 | 2.202 |
| North | 32 | Recovery | Q40 | 2.661 | 155.619 | 1.079 | 4.664 | 12.559 | 34684.36 | 2.154 |
| North | 32 | Recovery | Q35 | 0.888 | 22.902 | 0.429 | 2.078 | 9.194 | 7576.91 | 0.677 |
| North | 32 | Recovery | Q34 | 1.565 | 33.920 | 0.505 | 2.138 | 11.869 | 7966.36 | 1.263 |
| South | 10 | 3h HS | Q05 | 0.759 | 0.698 | 0.091 | 0.249 | 0.513 | 3.82 | 0.873 |
| South | 10 | 3h HS | Q11 | 0.476 | 1.050 | 0.176 | 0.288 | 0.510 | 17.45 | 0.252 |
| South | 10 | 3h HS | Q06 | 0.379 | 0.690 | 0.205 | 0.106 | 0.708 | 19.09 | 0.469 |
| South | 10 | 3h HS | Q05 | 0.417 | 0.655 | 0.245 | 0.228 | 0.532 | 2.18 | 0.740 |
| South | 10 | 3h HS | Q06 | 0.616 | 1.031 | 0.269 | 0.106 | 0.799 | 63.27 | 0.581 |
| South | 10 | 3h HS | Q11 | 0.290 | 1.458 | 0.308 | 0.277 | 0.621 | 10.36 | 0.307 |
| South | 10 | Recovery | Q02 | 0.411 | 6.691 | 0.998 | 0.074 | 0.802 | 14.73 | 1.773 |
| South | 10 | Recovery | Q03 | 0.684 | 1.579 | 0.536 | 0.084 | 0.646 | 17.45 | 0.011 |
| South | 10 | Recovery | Q01 | 0.380 | 6.417 | 0.054 | 0.115 | 1.193 | 24.55 | 1.195 |
| South | 10 | Recovery | Q01 | 0.576 | 5.723 | 0.037 | 0.148 | 1.374 | 78.00 | 0.689 |
| South | 10 | Recovery | Q03 | 0.550 | 1.122 | 0.420 | 0.076 | 0.522 | 1.64 | 0.006 |
| South | 10 | Recovery | Q02 | 0.955 | 6.527 | 1.091 | 0.067 | 0.668 | 17.45 | 1.617 |
| South | 24 | 3h HS | Q36 | 3.619 | 1.154 | 0.909 | 0.789 | 11.758 | 72986.18 | 2.236 |
| South | 24 | 3h HS | Q15 | 3.204 | 0.565 | 1.338 | 1.119 | 9.021 | 29548.91 | 3.987 |
| South | 24 | 3h HS | Q14 | 3.400 | 24.570 | 0.130 | 2.414 | 20.755 | 14661.27 | 2.725 |
| South | 24 | 3h HS | Q36 | 4.439 | 1.997 | 1.066 | 1.128 | 10.064 | 68667.82 | 2.614 |
| South | 24 | 3h HS | Q14 | 4.353 | 36.388 | 0.202 | 2.622 | 22.000 | 15945.82 | 3.254 |
| South | 24 | 3h HS | Q15 | 3.122 | 0.768 | 1.189 | 1.094 | 7.750 | 25458.00 | 4.203 |
| South | 24 | Recovery | Q08 | 0.855 | 1.233 | 0.826 | 0.286 | 1.495 | 3514.91 | 5.829 |
| South | 24 | Recovery | Q09 | 1.095 | 2.963 | 0.530 | 0.432 | 1.319 | 4135.64 | 0.823 |
| South | 24 | Recovery | Q07 | 0.629 | 1.755 | 0.674 | 0.218 | 1.077 | 10233.82 | 2.903 |
| South | 24 | Recovery | Q07 | 0.598 | 1.029 | 1.125 | 0.194 | 0.751 | 7940.18 | 2.521 |
| South | 24 | Recovery | Q09 | 1.466 | 3.331 | 0.680 | 0.386 | 1.305 | 3708.00 | 0.697 |
| South | 24 | Recovery | Q08 | 0.670 | 1.700 | 0.946 | 0.347 | 1.726 | 3396.00 | 7.140 |
| South | 28 | 3h HS | Q31 | 1.408 | 51.397 | 0.578 | 0.978 | 11.149 | 80153.45 | 3.539 |
| South | 28 | 3h HS | Q33 | 3.925 | 2.492 | 0.857 | 2.118 | 21.575 | 48360.55 | 3.224 |
| South | 28 | 3h HS | Q32 | 2.495 | 16.000 | 0.858 | 1.691 | 20.599 | 63786.55 | 0.000 |
| South | 28 | 3h HS | Q31 | 1.500 | 82.241 | 0.680 | 1.360 | 12.579 | 74359.64 | 5.270 |
| South | 28 | 3h HS | Q32 | 2.941 | 21.665 | 1.115 | 1.214 | 22.721 | 57357.82 | 0.000 |
| South | 28 | 3h HS | Q33 | 3.604 | 2.679 | 0.630 | 1.981 | 18.209 | 44668.36 | 3.391 |
| South | 28 | Recovery | Q20 | 2.082 | 2.101 | 0.148 | 0.399 | 1.370 | 24670.36 | 7.704 |
| South | 28 | Recovery | Q21 | 1.602 | 0.687 | 0.423 | 0.383 | 2.428 | 21434.18 | 1.821 |
| South | 28 | Recovery | Q19 | 2.553 | 1.270 | 0.919 | 0.611 | 2.984 | 17653.64 | 3.443 |
| South | 28 | Recovery | Q19 | 1.785 | 1.260 | 1.003 | 0.611 | 4.644 | 21844.91 | 2.725 |
| South | 28 | Recovery | Q21 | 1.896 | 0.711 | 0.434 | 0.320 | 2.147 | 20472.00 | 2.004 |
| South | 28 | Recovery | Q20 | 2.101 | 2.341 | 0.226 | 0.370 | 1.319 | 23473.09 | 7.716 |
| South | 32 | 3h HS | Q16 | 0.544 | 82.696 | 0.572 | 0.238 | 4.350 | 6226.91 | 0.001 |
| South | 32 | 3h HS | Q18 | 0.710 | 55.574 | 0.411 | 0.937 | 7.714 | 21591.82 | 0.000 |
| South | 32 | 3h HS | Q17 | 0.440 | 56.578 | 0.386 | 0.297 | 3.870 | 21061.64 | 1.516 |
| South | 32 | 3h HS | Q16 | 0.818 | 111.534 | 0.585 | 0.364 | 5.551 | 3784.91 | 0.002 |
| South | 32 | 3h HS | Q17 | 0.521 | 69.930 | 0.558 | 0.412 | 4.933 | 20457.82 | 1.899 |
| South | 32 | 3h HS | Q18 | 0.566 | 65.828 | 0.627 | 0.778 | 7.094 | 23208.55 | 0.000 |
| South | 32 | Recovery | Q10 | 1.998 | 174.999 | 1.360 | 0.548 | 6.090 | 12600.00 | 10.140 |
| South | 32 | Recovery | Q12 | 1.080 | 71.963 | 0.912 | 1.203 | 4.788 | 17305.64 | 1.445 |
| South | 32 | Recovery | Q04 | 1.848 | 237.213 | 0.709 | 0.810 | 5.765 | 26970.00 | 0.245 |
| South | 32 | Recovery | Q04 | 1.574 | 280.615 | 0.822 | 1.253 | 5.138 | 30946.91 | 0.142 |
| South | 32 | Recovery | Q12 | 1.167 | 92.068 | 1.053 | 1.203 | 5.170 | 14163.82 | 1.872 |
| South | 32 | Recovery | Q10 | 1.614 | 171.342 | 1.253 | 0.275 | 5.100 | 11066.73 | 8,244 |
